# Natural variation *MeMYB108* associated with tolerance to stress-induced leaf abscission linked to enhanced protection against reactive oxygen species in cassava

**DOI:** 10.1007/s00299-022-02879-6

**Published:** 2022-05-24

**Authors:** Bin Wang, Shuxia Li, Liangping Zou, Xin Guo, Jiaxin Liang, Wenbin Liao, Ming Peng

**Affiliations:** 1 Institute of Tropical Bioscience and Biotechnology, Chinese Academy of Tropical Agricultural Sciences, Haikou, 571101 China; 2 Key Laboratory of Biology and Genetic Resources of Tropical Crops, Institute of Tropical Bioscience and Biotechnology, Chinese Academy of Tropical Agricultural Sciences, Haikou, China; 3 College of Life Sciences, Heilongjiang University, Heilongjing, 150080 China

**Keywords:** *MYB* transcription factor, Natural variation, Leaf abscission, Reactive oxygen scavengers, Drought, Cassava

## Abstract

**Key Message:**

Natural variation of the *MeMYB108* exon was associated with reactive oxygen scavengers led to alleviate leaf abscission under drought in cassava.

**Abstract:**

The reactive oxygen scavengers play important roles in regulating the cassava (*Manihot esculenta* Crantz) leaf abscission induced by stresses. To date, the relationship between natural variations of *MYB* genes and reactive oxygen scavengers under drought in cassava genotypes remains unclear. Here, we reported the transcription factor *MeMYB108* played an important role in regulating leaf abscission exposed to drought in cassava. The expression levels of *MeMYB108* in abscission zones of cassava leaf pulvinus were higher in cassava genotype SC124, which were less easy to shed leaves under stress than cassava genotype SC8 when the leaf abscission induced by the same drought condition. Compared with wild type and interference expression plants, overexpression of *MeMYB108* significantly reduced the drought-induced leaf abscission rate under drought. The consecutively 2-year analysis of reactive oxygen scavengers showed significant differences among different cassava genotypes under drought-induced leaf abscission, indicating the relevance between reactive oxygen scavengers and leaf abscission. Correlation analysis revealed the natural variation of the *MeMYB108* exon was associated with reactive oxygen scavengers during drought-induced leaf abscission. Association analysis between pairwise LD of DNA polymorphism indicated the *MeMYB108* allele enhanced the tolerance of cassava to drought-induced leaf abscission. Complementation transgenic lines containing the elite allele of *MeMYB108* ^SC124^ decreased the leaf abscission rate induced by drought conditions, demonstrating natural variation in *MeMYB108* contributed to leaf abscission tolerance induced by drought in cassava. Further studies showed *MeMYB108* played an active role in the tolerance of cassava to drought-induced leaf abscission by inducing scavenging of reactive oxygen species.

**Supplementary Information:**

The online version contains supplementary material available at 10.1007/s00299-022-02879-6.

## Introduction

Cassava is an important tropical crop with strong stress tolerance, high light efficiency and starch yield. The excellent stress tolerance of cassava is partly on the strength of the flexible leaf abscission mechanism. The leaf abscission and growth conversion under stress are key factors affecting the starch production of cassava photosynthesis (Liao et al. 2016a, b) Because cassava mainly grows in tropical and subtropical areas, how to deal with the seasonal drought resulting in leaf abscission is one of the leading problems in improving cassava starch production. The significance of reactive oxygen species (ROS) is proved in regulating leaf abscission in cassava under drought (Liao et al. 2016a), and an effective ROS clearance mechanism can delay leaf senescence and abscission zone formation in leaf petioles (Liao et al. 2016a). Active oxygen scavengers such as catalase (CAT), superoxide dismutase (SOD), and ascorbate peroxidase (APX) play an important role in regulating leaf abscission induced by stresses (Liao et al. 2016a, b; Xu et al. 2013a). Xu et al. (2013b) overexpressed two ROS scavenging enzymes, *MeCu/ZnSOD* and *MeCAT1*, in cassava, which greatly enhanced the ability of cassava to scavenge ROS, alleviating drought-induced leaf abscission in transgenic cassava compared with the wild-type controls (Xu et al. 2013a, b). Therefore, the ROS scavenging system exerts an important regulatory effect on the leaf abscission of cassava induced by drought.

A significant difference can be found in drought-promoted leaf abscission among different cassava genotypes (Oliveira et al. 2017; Zhao et al. 2015), which take different countermeasures to resist leaf abscission induced by drought. For example, cassava cultivar SC124 (drought-tolerant genotype with slight leaf abscission under drought), Arg7 and SC8 (drought-sensitive genotype with severe leaf abscission under drought) adopt two distinct strategies to stop the growth and maintain life to fall off old leaves under drought conditions (Zhao et al. 2015). This significant phenotypic difference should be closely related to the genotypes of cassava.

Several members of the *MYB* family have been reported to be involved in the abscission process and drought response (Liao et al. 2016b; Wang et al. 2014; Gubert et al. 2014; Li et al. 2017). *MYB* family AS1 was proved to be critical for the proper placement of the floral organ abscission zone and influence the timing of organ shedding (Li et al. 2017). The *Arabidopsis* homologous gene *AtMYB2* of *MeMYB108*, together with *AtMYC2*, is involved in regulating the drought stress response gene RD22 in the ABA-dependent signaling pathway (Abe et al. 2013). Supplementaryly, stress, including drought, can cause plant leaves to fall off. Many *MYB* members have been reported to participate in mechanisms to resist drought. For example, overexpression of *MYB12*, one of the regulatory genes of the flavonoid pathway, reduces water loss (Nakabayashi et al. 2014). *MYB60* is a negative regulator of drought stress (Oh et al. 2011). *MYB41*, *MYB88*, *MYB44* and *MYB102* are positive regulators of drought stress (Xie et al. 2010; Denekamp et al. 2003; Mengiste et al. 2003; Cominelli et al. 2008; Jung et al. 2008). *MYB2*, *MYB96* and *MYB15* are positive regulators of drought stress, attaching to the *MYB* binding site of the key genes such as RD22, thereby regulating ABA-dependent signaling pathways (Urao et al. 1996; Abe et al. 2013; Ding et al. 2009; Seo et al. 2009). *MYB2* is involved in the regulation of abiotic stresses such as drought stress, salt stress and phosphorus deficiency. Abe et al. reported that *AtMYB2* regulates RD22 under drought or salt stress conditions that lead to ABA synthesis. As a signaling molecule, ABA induces expression of bHLH-like proteins RD22, BP1 and At*MYB*2, which bind to drought stress response gene *RD22*. The expression of the *RD22* gene is regulated by the *MYC* and *MYB* binding sites of the promoter region (Abe 2013). Yoo et al. reported that *AtMYB2* directly regulates calmodulin and enhances the resistance of *Arabidopsis* to salt stress (Yoo et al. 2005). *AtMYB2* responds to a phosphorus deficiency signal and binds to the promoter region of the miR399f gene, regulating the expression of miR399f, thereby increasing the tolerance of *Arabidopsis* to phosphorus deficiency (Baek et al. 2013). In addition, *AtMYB2* also regulates whole plant senescence by inhibiting cytokinin-mediated branch growth at the late growth stage (Guo and Gan 2011).

The *MYB108* transcription factor contributes to the regulation of stamen maturation and male fertility in response to jasmonate signaling, which required for the correct timing of another dehiscence acting downstream of *MYB*21 in a transcriptional cascade. *MYB21* mediates stamen and pollen maturation and delays another dehiscence in response to jasmonate (Ajin et al. 2009). Rh*MYB*108, an R2R3-*MYB* transcription factor, is involved in ethylene- and JA-induced petal senescence and abscission in rose plants (Zhang et al. 2019). Taken together, these studies suggest that *MYB108* is involved in plant leaf abscission regulation. Our previous research reported the whole *MYB* superfamily genes in the cassava genome; many *MYB* family members respond to drought- and ethylene-induced leaf abscission. In this study, we reported that *MeMYB108* was more strongly expressed in SC124 (hard-to-shed genotype under stresses) than in SC8 (easy-to-shed genotype under stresses) with consistent drought-induced leaf abscission. Furthermore, we detailed the relationship between the natural variation of the *MeMYB108* gene and reactive oxygen scavengers with leaf abscission exposed to drought. We found that a total of 12 functional SNPs existed in the coding region of the *MeMYB108* gene and were correlated to CAT and SOD activities. Further studies confirmed that the reactive oxygen scavengers had ties to drought-induced leaf abscission. Correlation analysis of candidate genes showed that the natural variation of the *MeMYB108* exon was associated with drought-induced leaf abscission. For the transgenic cassava, the overexpression of *MeMYB108* significantly reduced the leaf abscission rate with drought-induced leaf abscission; by contrast, the interference expression of *MeMYB108* increased the leaf abscission rate. Phylogenetic analysis indicated that the *MeMYB108* allele might enhance the tolerance of cassava to drought-induced leaf abscission. Complementation transgenic lines containing the elite allele of *MeMYB108*^SC124^ decreased the leaf abscission rate induced by drought conditions, demonstrating that natural variation in *MeMYB108* contributed to leaf abscission tolerance in cassava induced by drought. Further studies showed that *MeMYB108* played an active role in the tolerance of cassava to drought-induced leaf abscission by inducing the scavenging of ROS.

## Materials and methods

### Plant material and treatments

A total of 97 cassava materials used in this research came from different countries and regions (See Supplementary Table S1 for detailed material names and sources). Cassava varieties SC124 and SC8 were used for RT-qPCR analysis of *MeMYB108* transcript levels with different leaf abscission induced by various stresses and hormone treatments. For understanding the *MeMYB108* responses with different degrees of drought-induced leaf abscission, three descriptions with different degrees of drought-induced leaf abscission were applied in this study, SLA means only the lower old leaf shed of cassava under drought, MLA indicated the middle and lower leaf abscission in cassava when suffered from drought condition, VLA mens upper, middle and lower leaf abscission shed induced by drought. To analyze the expression levels of *MeMYB108* under different stresses and phytohormone treatments, drought, ETH (100 μM), cold (4 °C), NaCl (200 mM), ABA (100 μM), and JA (100 μM) treatments were performed. Leaf abscission zones were harvested at 0, 0.5, 3, 9, 24, and 36 h after NaCl, ABA, ETH, and JA treatments and at 0, 3, 6, 9, 12, 15 and 18 days after dehydration treatment and at 0, 0.5, 1, 2, 3, and 4 days after cold treatment (Xiong et al. 2018).

### Gene subcellular localization

Methods and processes for subcellular localization of cassava genes were described as previously. The gene expression vector was constructed and transformed into *A. tumefaciens* GV3101 strain. The transformation and infection experiments were carried out on tobacco leaves after 6-week growth. The fluorescence signal was observed by confocal microscope (Olympus FV1000) after the 2-day transformation (Xiong et al. 2018).

### Phylogenetic analysis

The neighbor-joining method and bootstrap analyses (1000 replicates) were carried out for the phylogenetic tree constructed by MEGA6 software. The visualization of the phylogenetic tree was realized using the online tool Evolview (Zhang et al. 2012). Multiple sequence alignment was performed using ClustalW.

### The reactive oxygen scavengers detected with drought-induced leaf abscission and phenotypes investigation in cassava

A total of 97 cassava genotypes were cut into stem segments with uniform length, planted in flowerpots with an equal amount of soil and sand mixed uniform medium, and cultured in the rainproof shed. Cassava growing for 100 days was selected for the experiment. The treatment group stopped being watered for 12 days, and the control group was watered regularly every day. Without being water for 12 days, cassava genotypes showed drought-induced leaf abscission to different extents, leaves were collected from the upper, middle and lower parts of three plants, and roots of three plants were mixed and frozen in liquid nitrogen and stored at − 80 °C in the refrigerator. Detection was performed on the physiological phenotypes, including SOD activity, POD activity and CAT activity. Four physiological phenotypes were detected in Suzhou Keming Biotechnology Co., Ltd. The SOD activity was determined using the nitroblue tetrazolium reduction method and the NBT method. The POD activity was detected using the guaiacol method. The CAT activity was examined by the H_2_O_2_ ultraviolet absorption method following the corresponding reagent box operation instruction of Suzhou Keming company. Leaf abscission experiments exposed to drought were conducted in 2014 and 2015, and data of 2 consecutive years were obtained.

### Descriptive statistical analysis and average value comparison of reactive oxygen scavengers with drought-induced leaf abscission in cassava

SPSS18.0 software (http://www-01.ibm.com/software/analytics/spss/) (Jin et al. 2021) was used to conduct descriptive statistical analysis of phenotypic measurements and significant differences between the overall mean values of the drought-induced leaf abscission treatment group and the control group. Descriptive statistical analysis involved the maximum, minimum, mean, standard deviation and variation coefficient. One-way ANOVA was used to compare the mean values of F and P between the treatment and control groups. The 2-year values were converted into the drought-induced LAC coefficient according to Formula 1 (Bouslama et al. 1984):

$${\text{LAC = }}\frac{{\text{value of physiological characters in treatment group}}}{{\text{value of physiological characters in control group}}} \times 100$$

SPSS18.0 software was used for descriptive statistical analysis to analyze the maximum, minimum, average, standard deviation and variation coefficient of the drought tolerance of leaf abscission.

### Analysis of gene structure and conserved domain

The DNA sequences and CDS sequences of the cassava *MeMYB108* gene were obtained from the JGI cassava genome database (https://phytozome.jgi.doe.gov) (Liao et al. 2016a, Liao et al. 2016b) and imported into NCBI’s Splign tool (https://www.ncbi.nlm.nih.gov/sutils/splign/splign.cgi?textpage=online&level=form) (Liao et al. 2016a, b) to analyze the length, number and starting position of exons and introns of the gene. The GSDs (http://gsds.cbi.pku.edu.Cn/) (Hu et al. 2015) diagram of the gene structure was used. The CDS sequences of the *MeMYB108* gene were introduced into the NCBI’s CDD (https://www.ncbi.nlm.nih.gov/Structure/cdd/wrpsb.cgi) (Liao et al. 2016a, b) for sequence alignment and conserved structure analysis.

### Re-sequencing of the gene region

The natural variation and drought tolerance of *Memyb108* gene in 97 cassava genotypes were re-sequenced. The gene region of *MeMYB108* was re-sequenced using TA clone sequencing. Primers covering the entire length of the gene region were designed based on the DNA sequence of the cassava *MeMYB108* gene, and the primer sequences are shown in Supplementary Table S2. The genomic DNA of the population consisting of cassava materials was used as a template. PCR amplification was performed using TAKARA’s PrimerStar high-fidelity enzyme amplification, amplification protocols and procedures. The PCR product was subjected to agarose gel electrophoresis, gelatinization, and purification. The T vector was connected, and E. coli was transformed. A single clone was picked for PCR detection and sequencing. Finally, 97 DNA sequences of the *MeMYB108* gene of the cassava genotype were obtained. The BWA software aligned the sequences to the cassava genome version 6.1 (https://phytozome.jgi.doe.gov/pz/portal.html#!info?alias=Org_Mesculenta (Bredeson et al. 2016), SNPs were analyzed GATK software (V3.8–0, https://software.broadinstitute.org/gatk/) (Schilbert et al. 2020) for calling and low-quality SNP filtering. The multiplex PCR-targeted resequencing work was completed in Shanghai Wing and Applied Biotechnology. The SNP information is imported into the snpEff software (http://snpeff.sourceforge.net/SnpEff_manual.html) (Li et al. 2020), and the obtained SNPs are functionally annotated using the cassava genome version 6.1 sequence and annotation information.

### Nucleotide diversity analysis

The DNA sequence of the *MeMYB108* gene of cassava material was introduced into MEGA4.0 software (Tamura et al. 2007) for sequence alignment. The saved file of MEG format was opened using DnaSP5 software (Librado et al. 2009) to analyze the nucleoside acid diversity, statistical type and the number of SNP variants.

### Haplotype analysis

The *MeMYB108* haplotype was extracted using the DnaSP5 software and saved in NRF format. The haplotype network of the *MeMYB108* gene was mapped using NETWORK5 (http://www.fluxus-engineering.com) (Lin et al. 2017), and the sequence of the haplotype was introduced into MEGA4.0 to draw a phylogenetic tree. Linkage disequilibrium between the polymorphic sites of the two *MYB* genes was analyzed using Haploview version 4.2 software (Barrett et al. 2005).

### Candidate gene association analysis

According to the sequencing results, the SNP locus and genotyping of cassava material were extracted and imported into TASSEL2.1 soft (Yu et al. 2006), combined with drought tolerance-related phenotypic drought tolerance coefficient as a phenotype. Genome-wide EST-SSR marked the population structure Q-value matrix and family effect values as covariance for candidate gene association analysis. The significance association threshold was recorded as *P* < 0.05, and the *P* value was controlled by FDR (Benjamini and Hochberg 1995). The *P*_FDR < 0.05 was considered to be significantly associated with the phenotype.

### Plasmid construction and cassava transformation

The plasmid construction for *MeMYB108* transgenesis in cassava was carried out as described previously (Zhang et al. 2013). The expression cassette of cassava *MeMYB108* was inserted into the binary vector pCAMBIA1301 under the control of the CaMV 35S promoter to generate 35S::*MeMYB108* containing the hygromycin phosphotransferase gene (*hpt*). The construct was introduced into *A. tumefaciens* strain LBA4404 and then used for genetic transformation. The embryogenic callus of cassava TMS60444 and cassava SC8 and *A. tumefaciens*-mediated genetic transformation were performed as described by Xu et al. (2013a, b). To construct the RNAi plasmid, a 212-bp cDNA fragment of *MeMYB108* was amplified from cassava and inserted into the vector pCAMBIA1301 as described previously (Wang et al. 2004). For the complementation test, the coding region of *MeMYB108* was amplified from SC124 to obtain the gene fragment, which was inserted into the pCAMBIA1301 vector to generate a pCAMBIA1301::*MeMYB108-*SC124 expression cassette. The primers are listed in Supplementary Table S2. The cloned constructs were transformed into *A. tumefaciens* strain LBA4404 cells and transferred into cassava as described previously (Xu et al. 2013a, b).

## Results

### *MeMYB108* is associated with drought-induced leaf abscission in cassava

**Fig. 1:**
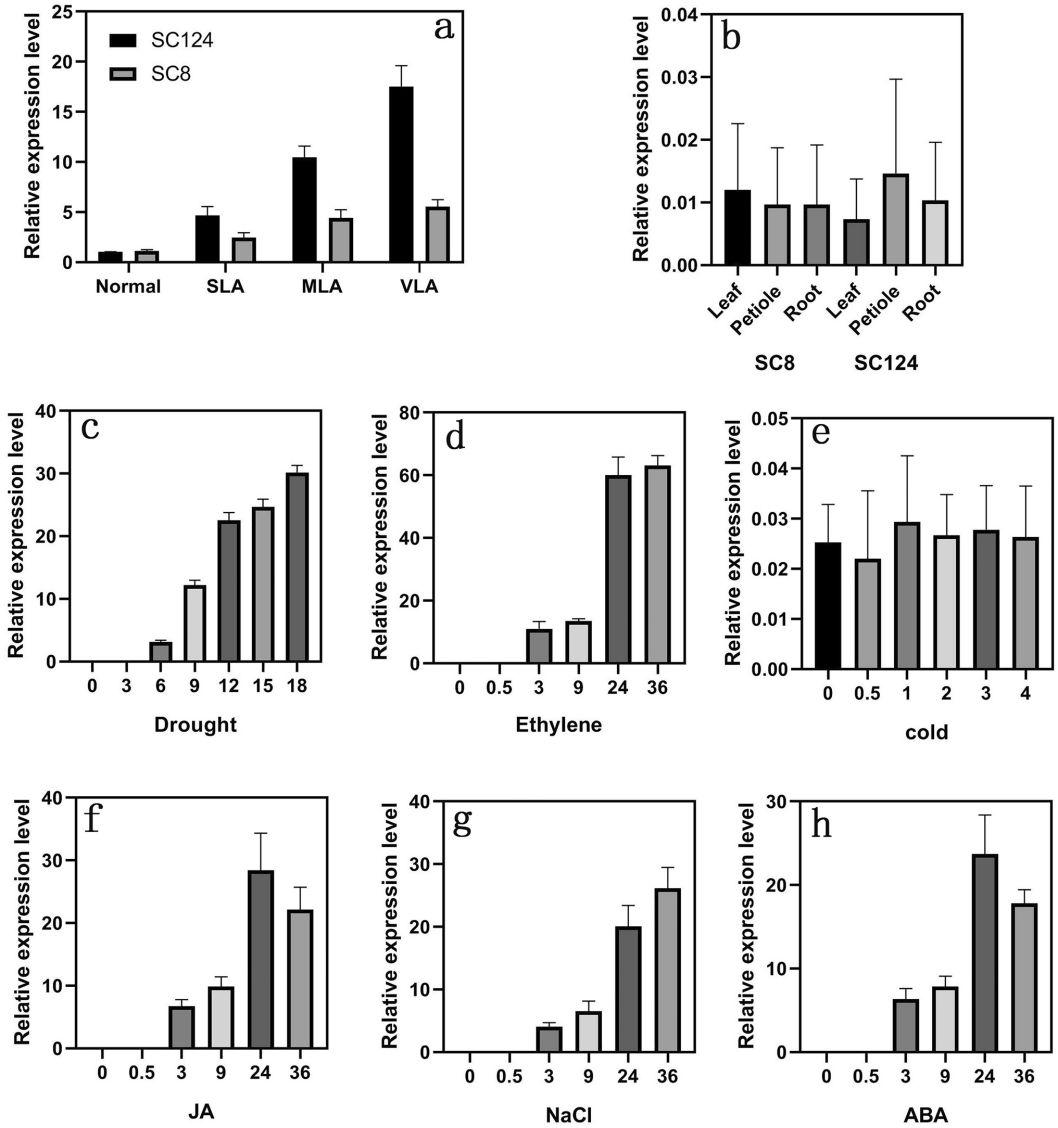
*MeMYB108* is associated with leaf abscission in cassava. **a** RT-qPCR analysis of *MeMYB108* in cassava genotype SC124 and SC8 with the same leaf abscission exposed to drought. Normal: No leaf abscission exposed to normal water condition; SLA: slight leaf abscission exposed to drought; MLA: moderate leaf abscission exposed to drought; VLA, very serious leaf abscission exposed to drought. **b** *MeMYB108* expression levels were detected in the leaf, petiole and root in both cassava genotype SC124 and SC8 under the same normal watering condition. **c** and **d** *MeMYB108* expression levels were analyzed during leaf abscission promoted by drought (**c**) and ethylene (**d**) in cassava SC124. **e–h** Expression levels of *MeMYB108* under various abiotic stresses and hormone treatments in cassava SC124. The cassava plants were subjected to cold (4 °C) (**e**), JA (100 μM) (**f**), NaCl (200 mM) (**g**), ABA (100 μM) and (**h**) treatments. All samples were collected from 100-day-old cassava plants, and only abscission zones of cassava were sampled for analysis. Data in the graphs are presented as means ± SE based on four technical replicates

Our previous work confirmed that the leaf abscission of cassava had obvious difference in different cassava genotypes under the same drought condition (Zhao et al. 2015; Liao et al. 2016a, b). To figure out the response of *MeMYB108* under drought-induced leaf abscission condition in different cassava genotypes, the expression patterns of *MeMYB108* were investigated in SC124 and SC8, which represented leaf abscission to a different extent under the same drought condition (Oliveira et al. 2017; Zhao et al. 2015). Specifically, we analyzed the *MeMYB108* expression patterns in the samples of the leaf abscission zones, covering no leaf abscission under normal water conditions (NLA), as well as slight leaf abscission (SLA), moderate leaf abscission (MLA) and very serious leaf abscission (VLA) induced by drought with quantitative PCR. The results showed that the expression of *MeMYB108* in SC124 was higher under SLA and MLA conditions and more strongly induced under the VLA condition than that in SC8 (Fig. 1a). These results suggested that the expression of *MeMYB108* was significantly related to drought-induced leaf abscission in cassava.

### MeMYB108 is an MYB family member

The all of *MYB* gene sequences of cassava genotype AM560-2 were obtained from the JGI cassava genome database (Supplementary Fig. 1a). The *MeMYB108* gene region was 1027 bp long, including two introns (148 bp and 98 bp in length, respectively) and three coding exons (160 bp, 130 bp and 517 bp, respectively). The CDS sequence of the *MeMYB108* gene was 807 bp in length and encoded 268 amino acids. The CDS sequences were aligned in the conserved domain database (CDD) of NCBI. Two *MYB* binding domains were discovered in the N-terminus of *MeMYB108,* indicating the *MeMYB108* gene might belong to the R2R3 type *MYB* family. *MeMYB108* was confirmed as a nucleus-localized protein in *Nicotiana benthamiana* leaves by infiltrating with *Agrobacterium tumefaciens* (strain GV3101) containing 35S: *MeMYB108*-GFP (Supplementary Fig. 1b).

### Expression profiles of *MeMYB108* induced by various leaf abscission-promoting treatments in cassava

To determine the spatiotemporal expression patterns of *MeMYB108* under normal growth conditions, we isolated total RNA in three representative tissues (root, petiole, and leaf) from SC8 and SC124 genotypes and performed RT-qPCR analysis (Fig. 1b–h). The data indicate no expression of *MeMYB108* in all tested tissues under normal conditions (Fig. 1b); that is, *MeMYB108* is an inducible functional gene. Our previous research identified that some functional genes could be induced by environmental factors of leaf abscission promotion, such as ethylene, drought and cold. Moreover, the expression profiles of *MeMYB108* in response to those stress-induced leaf abscissions were carried out. To specify the relevance of the function of *MeMYB108* to leaf abscission, we only focused on its expression pattern in tissues of leaf abscission zones, which were identified to significantly increase in drought and ethylene-induced abscission (Fig. 1c, d) rather than in cold-induced abscission (Fig. 1e). Furthermore, we also found that the expression levels of *MeMYB108* in leaf abscission zones were strongly induced by other stresses, including JA (Fig. 1f), NaCl (Fig. 1g) and abscisic acid (Fig. 1h). All those results verified that *MeMYB108* could be strongly induced by stresses of leaf abscission promotion, suggesting the pertinence of the function of the *MeMYB108* with the stress-induced leaf abscission.

### Overexpression of *MeMYB108* in cassava decreases the VLA-induced leaf abscission rate

**Fig. 2:**
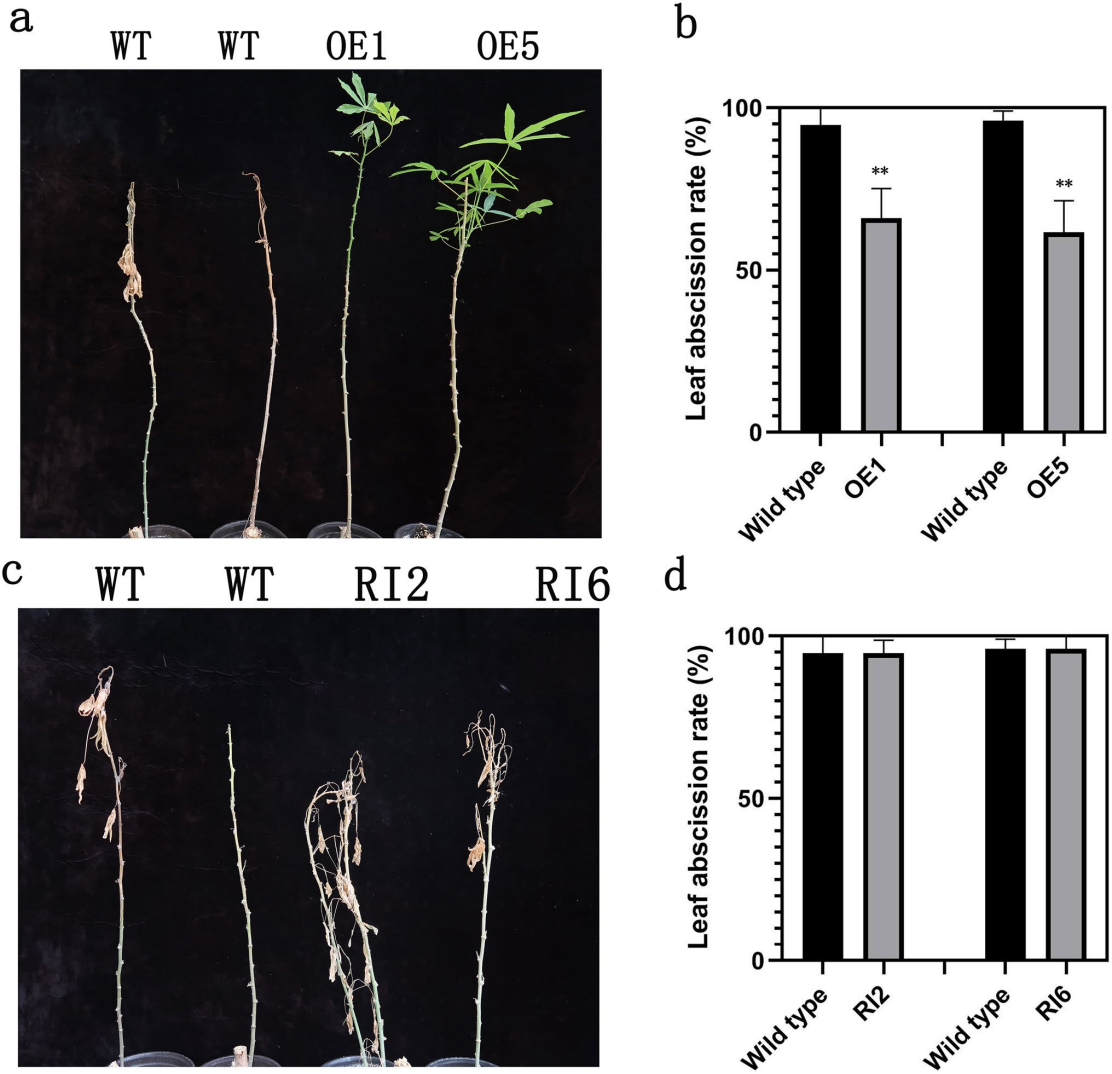
*MeMYB108* transgenic cassava plants indicated that *MeMYB108* expression contributed to alleviating leaf abscission exposed to drought. **a** *MeMYB108*-OE and wild-type plants were subjected to VLA condition with rewatering for 7 days after water withholding for 21 days. **b** Leaf abscission rates of transgenic and wild-type plants tested in a. **c** *MeMYB108*-RNAi and wild-type plants were subjected to VLA condition with rewatering for 7 days after water withholding for 21 days. **d** Leaf abscission rates of transgenic and wild-type plants tested in **c**. All samples were collected from 100-day-old cassava plants for analysis. Data in the graphs are presented as means ± SE based on four technical replicates. Asterisks indicated statistically significant differences calculated with Student’s *t* test: **P < 0.01. Bars = 50 mm

To understand whether the biological function of *MeMYB108* relates to the stress-induced leaf abscission, *MeMYB108* transgenic cassava plants of over-expression and RNA interference were obtained. The transgenic cassava lines were identified by hygromycin screening and real-time RT-PCR (Supplementary Fig. 2 a, b). The expression levels of *MeMYB108* in overexpression lines (OE1, OE4, OE5) were higher than those in wild-type and *MeMYB108*-RNA interference lines (RI2, RI5, RI6) (Supplementary Fig. 2a, b). Two transgenic lines (OE1 and OE5) and two RNA interference transgenic lines (RI2 and RI6) were selected for further analysis under drought-induced leaf abscission conditions. Under NLA conditions, no difference was found in growth between transgenic plants and wild plants (Supplementary Fig. 3). However, differences were significant under drought-induced leaf abscission conditions. The drought-induced leaf abscission rates in *MeMYB108*-OE lines, *MeMYB108*-RNA interference lines and wild types were measured under 7-day rewatering conditions after 21-day water withholding. Results indicated that only 49.2% to 74.6% of leaves were shed in *MeMYB108*-OE plants, while almost all leaves were shed in the wild plants (87.6–98.3%) and the *MeMYB108*-RNA interference lines (85.5–97.8%) (Fig. 2). For the leaf abscission rates almost the same under VLA conditions between wild plants and the *MeMYB108*-RNA interference lines, the leaf abscission rates of wild plants and the *MeMYB108*-RNA interference lines also compared under MLA conditions, the results showed almost all leaves were shed in the *MeMYB108*-RNA interference lines (89.8–97.9%), while wild plants (85.5–97.8%), while 76.7% to 86.4% of the wild-type plants shed their leaves under MLA conditions (Supplementary Fig. 2c, d). These results indicated that *MeMYB108* regulated the drought-induced leaf abscission in cassava.

### The levels of reactive oxygen scavengers vary significantly in different cassava genotypes with drought-induced leaf abscission

**Fig. 3:**
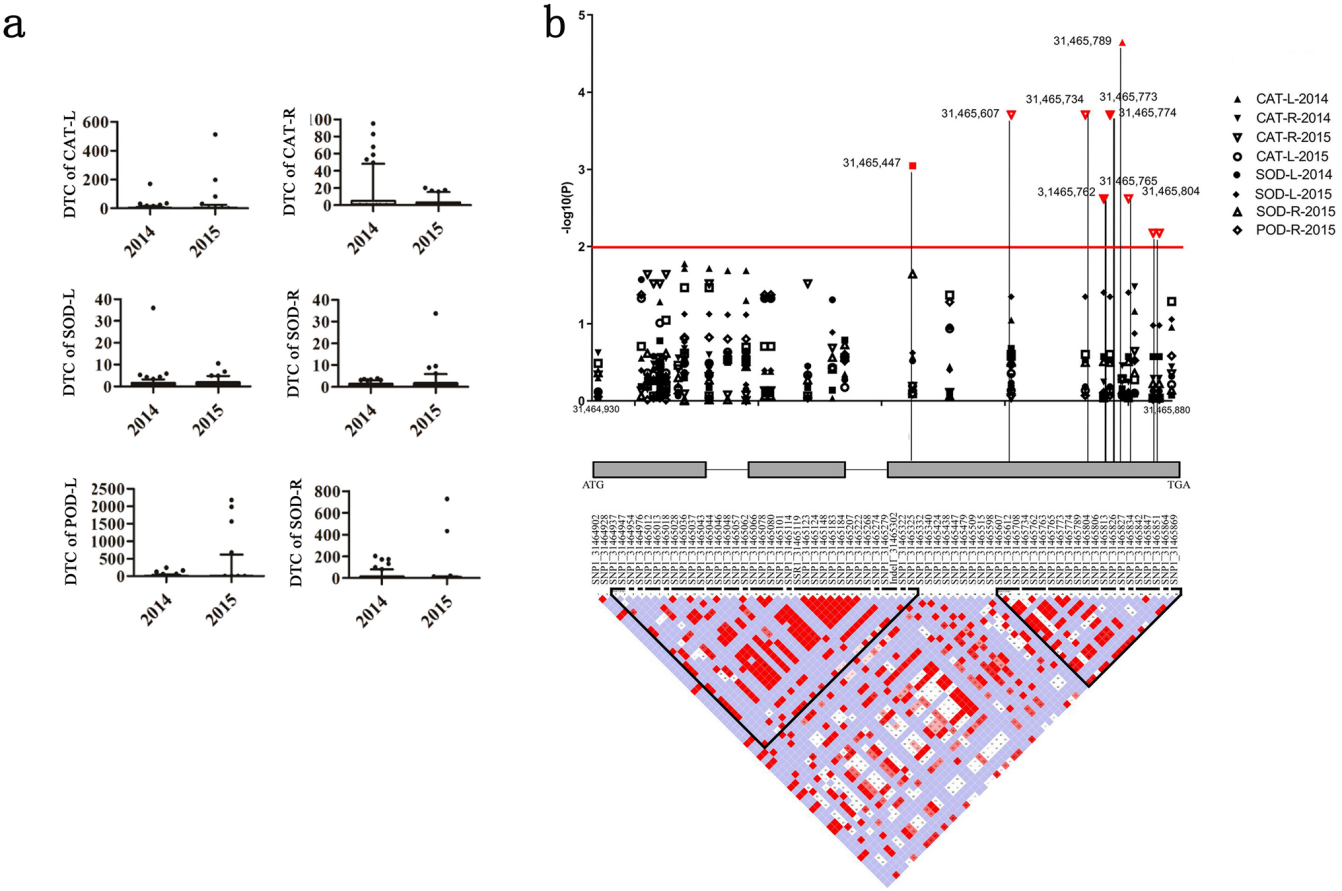
*MeMYB108* is associated with reactive oxygen scavengers during leaf abscission exposed to drought in cassava. **a** Reactive oxygen scavengers detected in consecutive 2 years varied significantly in different cassava genotypes during leaf abscission induced by drought in cassava. Cassava growing for 100 days was selected for the experiment. The treatment group was stopped watering for 12 days, and the control group was watered regularly every day. After 12 days of no watering, cassava genotypes showed different extents of leaf abscission exposed to drought. Leaves were collected from the upper, middle and lower parts of the four plants, and the roots of the four plants were mixed and frozen in liquid nitrogen and stored at − 80 °C in the refrigerator. The levels of CAT, CAD and POD activity in 97 cassava genotypes were detected during leaf abscission under drought conditions. L: leaves; R: roots. **b** The association analysis between pairwise LD of DNA polymorphisms in the *MeMYB108* was associated with reactive oxygen scavengers during drought-induced leaf abscission in cassava. A schematic of *MeMYB108* is shown on the x axis, and the significance of each variation associated with reactive oxygen scavengers with drought-induced leaf abscission is shown on the y axis. The SNPs with significant variation between genotypes are connected to the pairwise LD diagram with a solid line (Red line) (*P* < 0.01). Red triangles in the pairwise LD diagram highlight the strong LD of SNP1_31465734, SNP1_31465773, SNP1_31465774, SNP1_31465762, SNP1_31465765, and SNP1_31465804. The six SNPs are significantly associated with CAT (*P* < 0.01)

Our previous work demonstrated that ROS could regulate cassava leaf abscission under drought conditions. In view of this, we measured the levels of reactive oxygen scavengers (SOD, CAT, POD) in different cassava genotypes with consecutively 2-year drought-induced leaf abscission (2014 and 2015). Among 97 cassava genotypes, the SOD content was the highest (33.81) in 2015 and the lowest (0.10) in 2014. The CAT content was the highest (512.81) in 2015 and the lowest (0.00) in 2014. The POD content was the highest (2178.25) in 2015 and the lowest (0.00) in 2014. The results showed the differential natural changes of reactive oxygen scavengers in various cassava genotypes with identical drought-induced leaf abscission (Supplementary Table S3). As the statistical results shown in Supplementary Table S3, the LAC based on the leaf abscission of various reactive oxygen scavengers is a continuous quantitative character and changes obviously. The greatest coefficient of reactive oxygen scavengers is CAT-R-2014, while the smallest is SOD-R-2014. The largest coefficient of variation is CAT-L-2015 (Fig. 3a; Supplementary Table S3). The results showed that levels of reactive oxygen scavengers varied greatly in different cassava genotypes with drought-induced leaf abscission.

### Natural variation of *MeMYB108* is associated with reactive oxygen scavengers during drought-induced leaf abscission in cassava

To investigate whether the natural variation of *MeMYB108* is associated with reactive oxygen scavengers during leaf abscission induced by drought in cassava, we performed the candidate gene association analysis. The nucleotide diversity analysis of the *MeMYB108* gene was carried out in 97 cassava genotypes. The results are shown in Supplementary Table S4. The gene region includes 87 single nucleotide polymorphisms (SNPs) and 2 insertion/deletion polymorphisms (i.e., Insert/Deletion, Indel). The exon region has a total of 59 SNPs, including 17 synonymous mutations and 42 non-synonymous substitutions; the latter results in 40 amino acid differences. The conserved domain includes 2 synonymous substitutions and 12 non-synonymous substitutions. The DNA nucleotide diversity was analyzed by the sliding window with a window size of 100 bp and a step size of 25 bp using DnaSP5 software. The results are shown in Supplementary Fig. 4. The average nucleotide diversity π value is 0.01322. In detail, the highest acid diversity is discovered in the first intron. A total of 87 SNPs and 2 Indels (including 18 base SSR insertion deletion and 1 single-base insertion deletion) have 38 sites with MAF greater than 5%.

The exon region of the *MeMYB108* gene includes 29 haplotypes and 7 major haplotypes (Supplementary Fig. 5). All these haplotypes can be divided into two categories (Supplementary Fig. 6). Three amino acid differences are discovered between the two categories, and positive selection is indicated in the third exon region (Supplementary Fig. 7). Category II may be derived from wild-type cassava W14, which may be introgressed into cassava cultivars during long-term breeding and produced by mutation in different directions. The linkage disequilibrium analysis of cultivars revealed two haplotypes existed in the nucleotide variation of the exon region of the *MeMYB108* gene, separated by recombination hotspots (Fig. 3b). The three SNPs that divide the haplotype into two major categories are located in haplotype 2, resulting in two amino acid variations (Fig. 3B).

To further understand the relationship between *MeMYB108* and drought-induced leaf abscission at the genetic level, we used the Haploview 4.2 software to analyze the missense mutations in related sites of the *MeMYB108* gene region. The block was divided into confidence intervals, statistical genes and traits. The haplotype consisting of functional SNP loci was associated with the leaf abscission tolerance coefficient (LAC), and the haplotype was correlated with the drought-induced leaf abscission.

The *MeMYB108* gene region includes 12 non-synonymous replacement SNPs significantly associated with drought-induced leaf abscission. The haplotype analysis of these 12 functional mutation sites was carried out, among which 9 SNPs constituted 1 monolithic block, including 5 haplotypes (frequency > 0.01). The cassava materials were grouped according to the 5 haplotypes, and a total of 96 haplotype groupings and drought-induced leaf abscission groupings were obtained. According to ANOVA analysis of the LAC of single traits among haplotype groups, *MeMYB108,* CAT-L-2014 (*P* = 2.0E−03), and POD-R-2015 (*P* = 9.0E−03) was significantly associated (Fig. 3a, Supplementary Table S5).

The correlation analyses between candidate gene *MeMYB108* and the drought-induced leaf abscission were conducted with the rare allelic variation filtered out under the condition of MAF > 0.05. The results are shown in Fig. 3b. A total of 38 SNP/Indel marker sites in the *MeMYB108* gene region are involved in the leaf abscission induced by drought-related phenotypic analysis. *MeMYB108* is significantly correlated with the drought tolerance coefficient of drought-induced leaf abscission of CAT, SOD, and POD under the threshold conditions (*P* < 0.05, FDR < 0.05). When *P* < 0.01, *MeMYB108* was significantly correlated with CAT. When *P* < 0.05, a total of 30 SNP loci and 1 intron SSR marker were significantly associated with drought-induced leaf abscission, 21 of which were located in exons, including 9 synonymous mutations and 12 non-synonymous mutations. The average interpretation rate of SNPs for phenotypic variation was 10.13%, ranging from 3.99 to 39.14%. Non-synonymous mutations were significantly associated with the drought tolerance coefficient of the drought-induced leaf abscission, such as CAT and SOD (Fig. 3b, Supplementary Table S6). Two groups of three SNPs with high linkage disequilibrium were indicated in the third exon region, located in the haplotype 2 interval. The first three SNPs are SNP1_31465734, SNP1_31465773 and SNP1_31465774, significantly associated with the drought tolerance coefficient of CAT and SOD. The second group of three SNPs was SNP1_31465762, SNP1_31465765 and SNP1_31465804, significantly relevant to CAT (Fig. 3b, Supplementary Table S6). The results showed that natural variations in *MeMYB108* had close ties to CAT with drought-induced leaf abscission.

### Genetic complementation test transferring the gene fragment of *MeMYB108* from SC124 to SC8 alleviates leaf abscission under the VLA condition

**Fig. 4:**
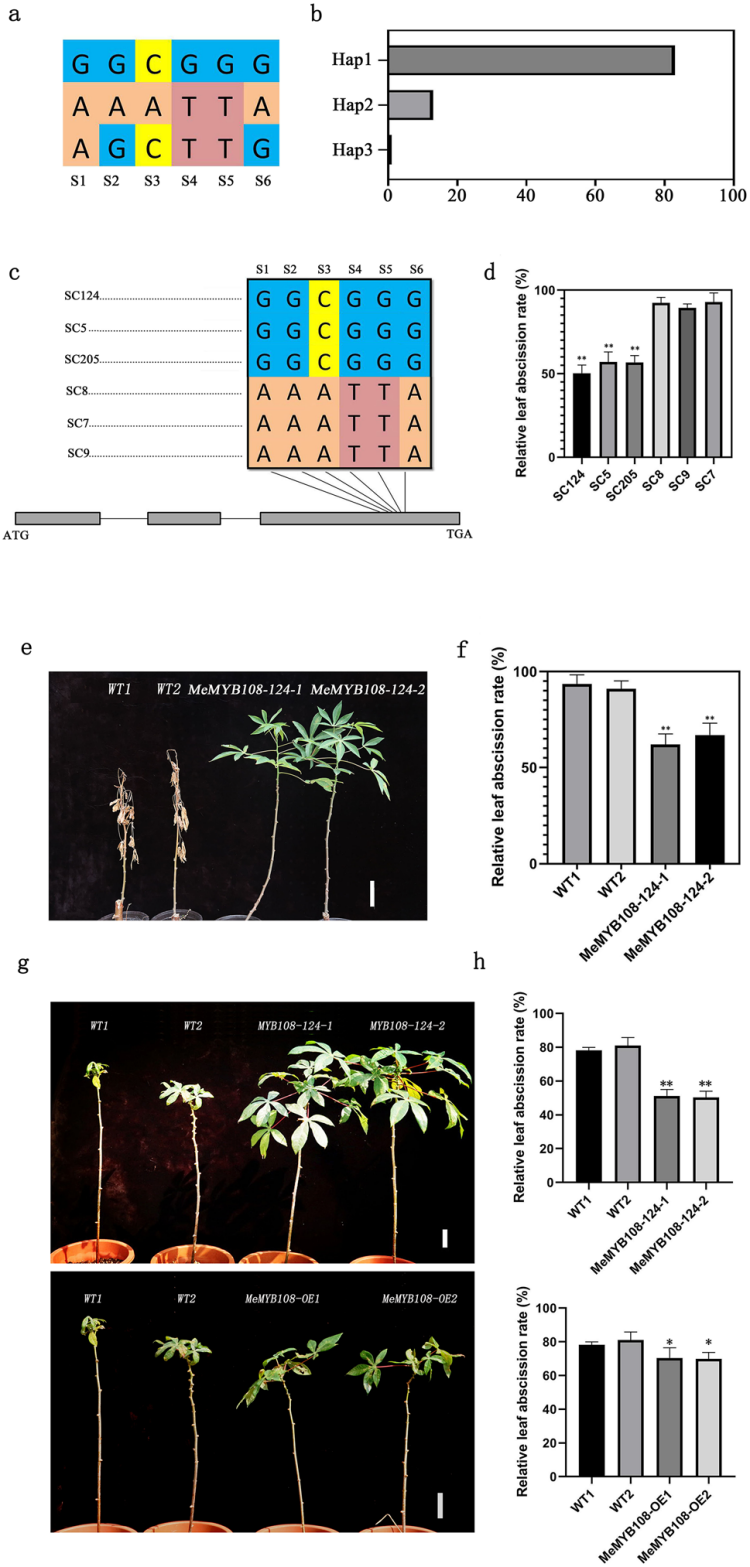
Gene complementation test of *MeMYB108* indicated that the elite alleles of *MeMYB108* alleviated leaf abscission exposed to drought in cassava. **a** Haplotype analysis of *MeMYB108* in 97 cassava accessions. S1 to S6 denote SNP1_31465734, SNP1_31465773, SNP1_31465774, SNP1_31465762, SNP1_31465765 and SNP1_31465804, respectively. **b** Haplotype analysis based on the phylogenetic tree of *MeMYB108* in 97 cassava genotypes was constructed based on six SNPs (S1-S6) (Supplementary Fig. 8). Significant linkage disequilibrium could be found among the six SNPs, and three haplotypes showed frequencies of 0.853, 0.134 and 0.013, respectively. **c** Haplotype analysis of *MeMYB108* in SC124, SC205, SC5 (the same alleles as SC124 at these six SNPs), SC8, SC7 and SC9 (the same alleles as SC8 at these six SNPs). **d** Leaf abscission rate analyses of *MeMYB108* in SC124, SC205, SC5, SC8, SC7 and SC9. Values are means ± SE (*n* = 4). Statistical significance was determined by Student’s t test. Asterisks indicated statistically significant differences calculated with Student’s *t* test: **P < 0.01. **e** Complementation test of *MeMYB108*-SC124 and wild-type (SC8) plants were subjected to VLA conditions without being watered for 28 days and then recovered for 7 days. Bars = 50 mm. **f** Leaf abscission rates of transgenic and wild-type plants tested in e. Values are means ± SE (*n* = 4). Statistical significance was determined by Student’s *t* test. **g** Comparsion of *MeMYB108*-SC124, *MeMYB108* overexpression and wild-type (SC8) plants were subjected to MLA conditions without being watered for 14 days and then recovered for 7 days. Bars = 50 mm. **h** Leaf abscission rates of transgenic (*MeMYB108*-SC124, *MeMYB108-OE*) and wild-type plants tested in **g**. Values are means ± SE (*n* = 4). Statistical significance was determined by Student’s *t* test. Asterisks indicated statistically significant differences calculated with Student’s t test: *P < 0.05, **P < 0.01

Six SNPs, i.e., SNP1_31465734, SNP1_31465773, SNP1_31465774, SNP1_31465762, SNP1_31465765 and SNP1_31465804, were significantly associated with CAT with drought-induced leaf abscission (Figs. 3b, 4a). The NJ cluster tree of 97 cassava genotypes/lines was constructed based on the six SNPs, and 97 cassava accessions were divided into two groups (Supplementary Fig. 8). Significant linkage disequilibrium can be found among the six SNPs, and three haplotypes exist with frequencies of 0.853, 0.134 and 0.013, respectively (Fig. 4b). To confirm the genetic effect of different alleles of *MeMYB108* on drought-induced leaf abscission of cassava, we selected the genotypes SC5 and SC205 (with identical alleles to SC124) and the genotypes SC9 and SC7 (with same alleles as SC8) at these six SNPs (Fig. 4c). We examined whether any difference exists in leaf abscission rates among these genotypes with the same drought-induced leaf abscission. The results indicated the leaf abscission rates of genotypes with the same allele as SC124 (SC5 and SC205) were significantly lower than those with the same allele as SC8 (SC9 and SC7) under VLA conditions (Fig. 4c, d). Only 46.7% to 63.2% of the plants with the same allele as SC124 wild-type shed their leaves, while almost 86.8% to 97.5% of the plants with the same allele as SC8 plants shed their leaves under VLA conditions (Fig. 4d).

To verify whether *MeMYB108* had the same allele as SC124 with drought-induced delayed leaf abscission in cassava, the genetic complementarity test of the *MeMYB108* gene fragment was conducted from SC124 to SC8 by transforming the fragment of *MeMYB108* of SC124 to SC8. We selected two independent lines expressing *MeMYB108* (*MeMYB108*-124). The results showed that under VLA conditions (28 days without watering and 7 days rewatering), the leaf abscission rate of *MeMYB108*-124 was lower than that of wild-type SC8 (Fig. 4e). Only 57.9% to 73.5% of the *MeMYB108*-124 plants and almost 87.3% to 98.9% of the wild-type plants shed their leaves under VLA conditions (Fig. 4f). The leaf abscission rate comparision also carried out between *MeMYB108*-124 and *MeMYB108* overexpression with the same wild type control under the MLA conditions, the results showed under MLA conditions, the leaf abscission rate of *MeMYB108*-124 was lower than that of *MeMYB108* overexpression plants and wild type (Fig. 4g). Only 46.2% to 55.5% of the *MeMYB108*-124 plants and almost 66.2% to 77.3% of the *MeMYB108* overexpression plants shed their leaves, while 76.7% to 86.4% of the wild-type plants shed their leaves under MLA conditions (Fig. 4f). These results supported that *MeMYB108* with the same allele as SC124 might help delay drought-induced leaf abscission in cassava.

### *MeMYB108-124* genetic complementation plants accumulate greater amounts of CAT in cassava under VLA conditions

**Fig. 5:**
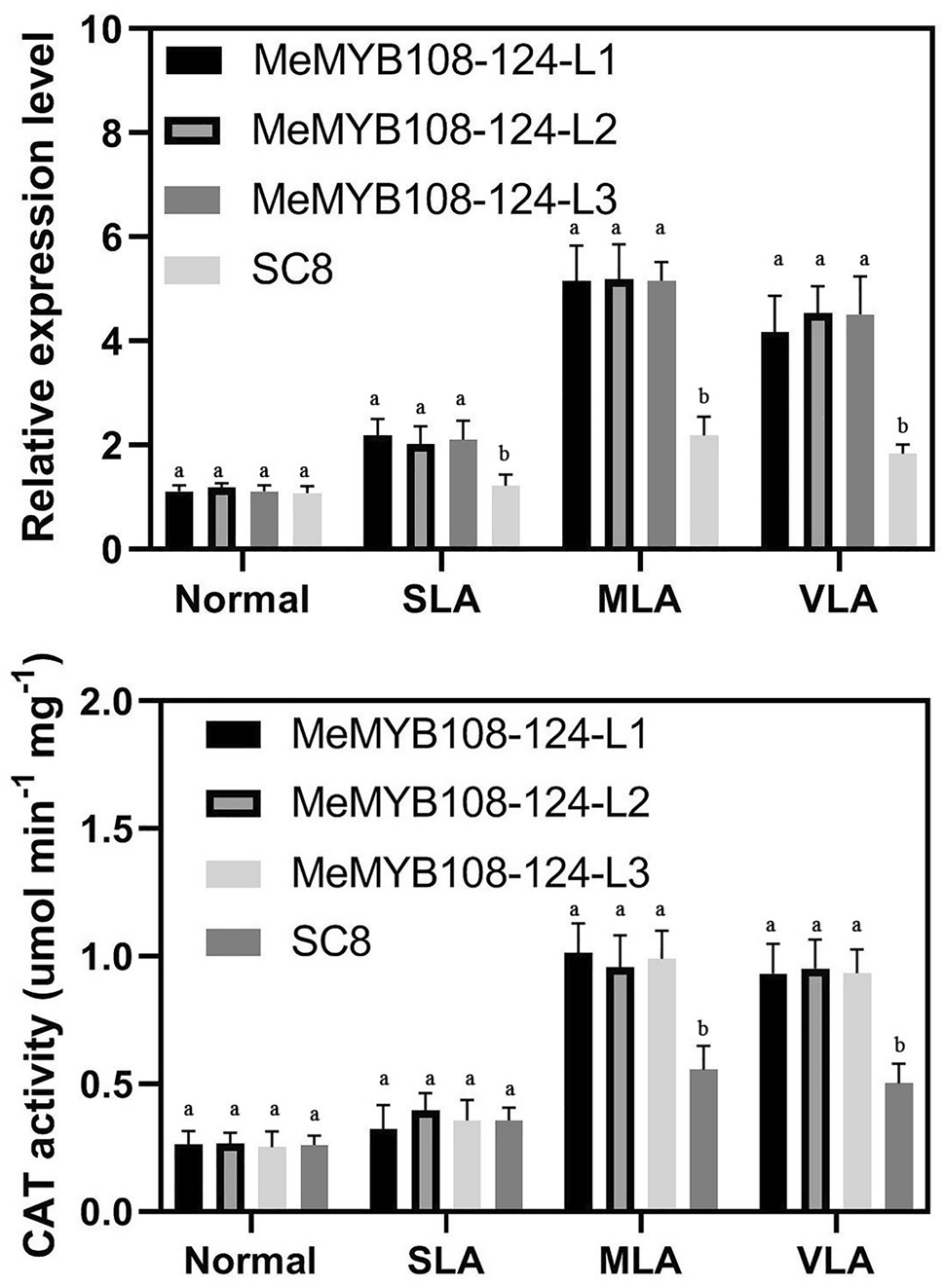
*MeMYB108*-124 genetic complementation lines accumulated greater amounts of catalase in cassava than in the wild-type plants under the same VLA condition **a** The *MeCAT1* expression levels in wild-type (SC8) and transgenic cassava *MeMYB108*-124 lines were carried out by qRT-PCR analysis under the same VLA condition. Total RNA was extracted from abscission zones, and the data were relative to the wild type, using actin7 as an internal control. Data are presented as means eight SD of four independent RNA samples. **b** Changes catalase activities between wild-type and transgenic cassava *MeMYB108*-124 lines under the same VLA condition. Data are presented as means eight SD from four independent measurements. Values labeled with different letters (**a** and **b**) at the same time point are significantly different by Duncan’s multiple comparison tests at *P* < 0.05.

The above study confirmed the relationship between the natural variation of *MeMYB108* and CAT. To further study whether that relation existed in the complementary line *MeMYB108-124*, the CAT level of *MeMYB108-124* was analyzed. We firstly analyzed the gene expression patterns of *MeMYB108*-124 and SC8 by qRT-PCR, covering the periods of drought-induced leaf abscission. In the three transgenic lines, the expression levels of *MeCAT* in *MeMYB108*-124 were significantly higher than that of wild type under VLA conditions and also than the wild-type SC8 under MLA and VLA conditions (Fig. 5a). This indicated that the natural variations of *MeMYB108* were obviously related to CAT levels in transgenic plant *MeMYB108*-124. Moreover, the CAT activities in wild-type SC8 and transgenic plant *MeMYB108*-124 were also analyzed. The total cellular CAT activities in wild-type and transgenic plant dramatically increased under VLA conditions compared with SLA conditions and remained at a high level under MLA conditions (Fig. 5b). The CAT activities were 0.224 and 0.66 under normal and MLA conditions, respectively, in the wild type but were 0.218 and 1.10, respectively, in the transgenic line. The differential CAT activities in the SC8 and transgenic line confirmed the important role of ROS scavengers in modulating drought-induced leaf abscission.

## Discussion

Transcription factors play important roles in regulating the physiological mechanism of plants under adverse environmental conditions (Khan et al. 2018). Several members of the *MYB* family in plants have been reported to modulate the abscission process (Liao et al. 2016b; Wang et al. 2014; Gubert et al. 2014; Li et al. 2017). AS1 activity is critical for the proper placement of the floral organ abscission zones and influences the timing of organ shedding (Li et al. 2017). The *MeMYB108* is a typical member of the *MYB* family and includes two *MYB* family DNA-binding domains in its protein amino acid sequence. The *MeMYB108* gene is classified into the R2R3-*MYB* subclass (Liao et al. 2016b). The *Arabidopsis* homologous gene *AtMYB2* of *MeMYB108*, together with *AtMYC2*, is involved in the regulation of the drought stress response gene RD22 in the ABA-dependent signaling pathway (Abe et al. 2013). Our analysis showed that *MeMYB108* was up-regulated when cassava suffered from drought-induced leaf abscission, indicating the positive role of *MeMYB108*. The expression patterns of *MeMYB108* under NLA, SLA, MLA and VLA conditions were analyzed by quantitative PCR. The results showed that under SLA, MLA and VLA, the expression of *MeMYB108* in SC124 was higher than that in SC8. These results suggested that the expression of *MeMYB108* might be related to the leaf abscission of cassava under drought stress.

In our previous studies, some functional genes regulating leaf abscission could be induced by environmental factors, such as ethylene, drought and low-temperature treatments (Liao et al. 2016a, b). Therefore, the present study analyzed the expression profiles of *MeMYB108* in those stress-induced leaf abscission treatments. The results showed that the transcriptional levels of *MeMYB108* could be induced significantly in drought and ethylene-induced abscission rather than in cold-induced abscission. Moreover, *MeMYB108* participated in the leaf abscission process. Subsequent transgenic cassava experiments confirmed that *MeMYB108* did regulate the drought-induced leaf abscission process.

The nucleotide sequence diversity of the *MeMYB108* gene region is very high, and an average of one SNP marker is included every 11.80 bp. Also, the average nucleotide diversity π value of the nucleotide in the gene region of *MeMYB108* is higher than that of the *Arabidopsis AtMYB2* gene (Kamiya et al. 2002). A total of 14 SNPs exist in the conserved domain of *MeMYB108*, of which 12 cause amino acid variation. This variation has been proved to affect the activity of the gene function (Wells et al. 2008).

Strong linkage disequilibrium can be found between SNPs in the *MeMYB108* gene region, and the SNP locus in the exon region constitutes a variety of haplotypes. At least one error leads to amino acid differences of haplotypes. Moreover, haplotypes can be divided into two categories, one of which represents the highest similarity to the *MeMYB108* sequence derived from the wild-type cassava W14. Nzuki et al. identified that on the resequencing of cassava cultivars and the W14 whole genome, a large fragment of W14 origin was introduced at 31 Mb at the end of chromosome 1 of some cultivars (Nzuki et al. 2017). This indicated that *MeMYB108* in some cassava cultivars was derived from W14.

SNP variation in gene exons is proved to change the gene function [30]. Analysis of the Ka/Ks of the *MeMYB108* gene region revealed that the third exon was strongly positively selected between the two major haplotypes, possibly due to the nucleotide variation of *MeMYB108* in this region against cassava, and the contribution of agronomic traits was in line with breeding objectives. The presence of three SNPs in the two haplotypes resulted in two different amino acids; Supplementaryly, these three SNP loci were significantly associated with the leaf abscission induced by drought phenotype, suggesting that the nucleotide variation of *MeMYB108* might result in various cassava genotypes with different drought-induced leaf abscission. The *MeMYB108* gene of interest has a very different evolutionary feature in the gene region. *MeMYB108* has a relatively long haplotype at the 5′ and 3′ ends of the gene region, including most polymorphic sites, corresponding to the DNA binding domain of *MYB* and the transcriptional self-activation domain. Further analyses suggested that six SNPs (SNP 131,465,734, SNP 131,465,773, SNP 131,465,774, SNP 131,465,762, SNP 131,465,765 and SNP 131,465,804) were found significantly associated with CAT activity with drought-induced leaf abscission (Fig. 3b). The results showed that the natural variation of *MeMYB108* in CAT activity was significantly correlated with the leaf abscission under drought conditions. Based on the 6 SNPs, the NJ cluster tree of 97 cassava genotypes/lines was constructed, and 97 cassava accessions were divided into 2 groups. Significant linkage disequilibrium existed among the six SNPs, and three haplotypes presented frequencies of 0.853, 0.134 and 0.013, respectively. Moreover, the leaf abscission degree of the cassava genotypes SC5 and SC205 with the same six SNPs with SC124 was significantly lower than that of SC9 and SC7 with the same six SNPs with SC8. The leaf abscission degree of SC5 and SC205 was significantly lower than that of SC9 and SC7. Gene complementation experiments showed that leaf abscission rates of plants with the same six SNPs with SC8 were higher than that of SC124 with the same drought-induced severe leaf abscission (Fig. 6b). Those results indicated the six SNPs had a strong correlation with leaf abscission under severe drought stress.

Our previous work confirmed that ROS regulated the cassava leaf abscission process (Liao et al. 2016a, b). To study the natural variations of reactive oxygen scavengers, the reactive oxygen scavengers (SOD, CAT and POD) levels of different cassava genotypes were measured with leaf abscission induced by drought for 2 consecutive years. The results showed different natural changes of reactive oxygen scavengers in various cassava genotypes with consistent drought-induced leaf abscission. Subsequent experiments confirmed that the natural variation of *MeMYB108* was associated with CAT in *MeMYB108-124* plants. Furthermore, the CAT activity in wild-type SC8 and transgenic plants *MeMYB108*-124 was also analyzed. Total cellular CAT activities in wild-type and transgenic plants dramatically were greater under VLA conditions than SLA conditions and remained at a high level under MLA conditions. These data suggested that the expression of *MeMYB108-124* increased the activity of CAT, which decreased the accumulation of ROS in plants and enhanced the ability of cassava to resist leaf abscission under drought stress.

In this study, the *MeMYB108* gene was significantly associated with leaf abscission induced by drought tolerance coefficients of multiple drought tolerance traits. To further analyze the relationship between the *MeMYB108* gene and drought tolerance traits, haplotype association analysis was also performed on the functional SNP-related sites of the *MeMYB108* gene. The monomer domains consisting of functionally related sites were located in the transcriptional autoactivation domain of the *MYB* gene, indicating that the *MeMYB108* gene might have an allele of transcriptional autoactivation activity in natural variation. Moreover, the haplotype association analysis between *MeMYB108* and individual traits showed significant differences between one and three drought tolerance traits among various haplotypes.

AraNet functional interaction analysis indicated that *MeMYB108,* CAT, SOD, and POD might be in the same functional network with the help of some intermediate genes such as *Hop3*, *GBF3*, *MYB102*, *CPK32,* etc. (Supplementary Table S7). The *MeMYB108* had directly functional interactions with the SOD and CAT enzyme-encoding gene to participate in the formation of these physiological phenotypes, which regulated the active oxygen scavenging mechanism and osmotic adjustment under drought stress.

Gene function and regulation are complex relationship, involving many metabolic pathways, especially for transcription factors. *MeMYB108* is an induced gene, which can be induced to express by drought and hormones, suggesting that *MeMYB108* may be involved in regulating drought and hormone pathways, these pathways also regulate ROS production and ROS induced leaf abscission, the expression analysis we have done is more important to show that the gene can be induced by some adverse factors, and these factors are also related to the production of ROS. ROS and ROS scavengers production itself is synchronous, there is also a seesaw effect in their regulatory relationship. In the case of *MeMYB108*, the regulation of *MeMYB108* by some abscission stimulating factors and the natural variation of *MYB108* and the active oxygen scavengers may seem contradictory, but in fact they may be uniform, the continuous production of ROS during leaf abscission may induce the expression of *MeMYB108* and regulate the production of ROS scavengers, which may be the reason why *MeMYB108* is induced by some abscission stimulators, different natural cassava populations may have different SNPs in the exon of *MeMYB108* that lead to the production of different intensity of scavengers, and from this respect, the two aspects are uniform. This conclusion was also confirmed by *MeMYB108* gene expression pattern in different degrees of drought-induced leaf abscission in two different cassava germplasms. The expression levels of *MeMYB108* in abscission zones of cassava leaf pulvinus were higher in cassava genotype SC124, which were less easy to shed leaves under stress than cassava genotype SC8 when the leaf abscission induced by the same drought condition. Further studies showed *MeMYB108* played an active role in the tolerance of cassava to drought-induced leaf abscission by inducing scavenging of reactive oxygen species, natural variation in *MeMYB108* contributed to leaf abscission tolerance induced by drought in cassava.

The present study revealed that *MeMYB108* was more strongly induced in cassava genotype SC124, of which leaves were less easy to shed under stress than those of SC8 with identical drought-induced leaf abscission. The results showed that the natural variation of reactive oxygen scavengers in leaves and roots of 97 cassava genotypes with leaf abscission promoted by drought analyses was very different from those of other cassava genotypes. Some natural variations existed in the *MeMYB108*, which might be involved in regulating the reactive oxygen scavengers (SOD and CAT) changes under stress. Re-sequencing analysis of 97 cassava genotypes indicated that the *MeMYB108* gene region included 87 single nucleotide variants (SNPs) and two insertion/deletion variants (Insert/Deletion, Indel). The SNP locus of the coding region of the *MeMYB108* gene could be divided into 29 haplotypes, including 7 major ones. These seven major haplotypes could be classified into two categories, one of which was more closely related to wild cassava genotype W14, indicating that *MeMYB108* was positively selected during cassava breeding. Correlation analysis between *MeMYB108* and drought tolerance showed that 12 functional SNPs in the *MeMYB108* coding region were significantly associated with CAT activity, SOD activity and other traits. Moreover, the prediction of interaction with key genes of drought tolerance confirmed that *MeMYB108* and key genes involved in CAT and SOD could interact through intermediate metabolic pathways. Correlation analysis of candidate genes showed that the natural variation of the *MeMYB108* exon was associated with drought-induced leaf abscission. Transgenic cassava showed that overexpression of *MeMYB108* significantly reduced the leaf abscission rate, while the interference expression of *MeMYB108* increased the leaf abscission rate with drought-induced leaf abscission. Phylogenetic analysis indicated that the *MeMYB108* allele might enhance the tolerance of cassava to drought-induced leaf abscission. Complementation transgenic lines containing the elite allele of *MeMYB108*-SC124 showed decreased leaf abscission rate induced by drought conditions, demonstrating that natural variation in *MeMYB108* contributed to leaf abscission tolerance induced by drought in cassava. Further studies showed that *MeMYB108* played an active role in tolerating drought-induced leaf abscission in cassava by inducing the scavenging of ROS.

## Supplementary Information

Below is the link to the electronic supplementary material.

Supplementary file1 (XLSX 12 KB)

Supplementary file2 (DOCX 17 KB)

Supplementary file3 (DOCX 17 KB)

Supplementary file4 (DOCX 16 KB)

Supplementary file5 (DOCX 17 KB)

Supplementary file6 (DOCX 22 KB)

Supplementary file7 (DOCX 17 KB)

Supplementary file8 (DOCX 4029 KB)
