# Supplementary material for: Natural variation MeMYB108 associated with tolerance to stress-induced leaf abscission linked to enhanced protection against reactive oxygen species in cassava
Source: Plant Cell Rep. 2022 May 24;41(7):1573–87. doi: 10.1007/s00299-022-02879-6 (PMC9270272; doi:10.1007/s00299-022-02879-6)
Supplement: Supplementary file 2 — Supplementary file2 (DOCX 17 KB) [file 299_2022_2879_MOESM2_ESM.docx]

Supplementary Table S2. List of primers used in this study

| Primer | Forward primer (5’-3’) | Reverse primer (5’-3’) |
| --- | --- | --- |
| *MeMYB108* for sequence | ATGGACACTCAAGTAAGAAACCACG | AATCAGTTGCTGCTGTAGAAACCAAA |
| *MeMYB108* for OE expression and gene complementation test | ATGGACACTCAAGTAAGAAACCA | TCAAATCAGTTGCTGCTGTAGAA |
| *MeMYB108* for RNAi consruct | TGATATCCCTCCAAGTAGTGAAGC | GTGTCTCCATTTCCACCCAC |
| *MeMYB108* for qPCR | CCCAAGTTTCTTTGGTGTCAAG | CATTTCCACCCACCTGTTAGA |
| *MeACT7* for qPCR | ACAGAGAGAAGATGACCCAAATC | CCATCACCAGAATCCAGTACAA |
| *MeMYB108* for nucleus-localization analysis | ATGGACACTCAAGTAAGAAACCA | AATCAGTTGCTGCTGTAGAAACC |
