# Supplementary material for: Natural variation MeMYB108 associated with tolerance to stress-induced leaf abscission linked to enhanced protection against reactive oxygen species in cassava
Source: Plant Cell Rep. 2022 May 24;41(7):1573–87. doi: 10.1007/s00299-022-02879-6 (PMC9270272; doi:10.1007/s00299-022-02879-6)
Supplement: Supplementary file 3 — Supplementary file3 (DOCX 17 KB) [file 299_2022_2879_MOESM3_ESM.docx]

**Supplementary Table S3.** Summary of the variation of reactive oxygen scavengers in leaf and roots of different cassava varieties under leaf abscission exposed to drought in consecutive two years

| Traits | Organ | Year | Minimum | Maximum | Average | SD | CV(%) |
| --- | --- | --- | --- | --- | --- | --- | --- |
| SOD | Leaf | 2014 | 0.10 | 5.91 | 1.25 | 1.02 | 82.01 |
|  |  | 2015 | 0.22 | 10.58 | 1.71 | 1.57 | 91.72 |
|  | Root | 2014 | 0.16 | 3.84 | 1.11 | 0.75 | 67.93 |
|  |  | 2015 | 0.21 | 33.81 | 1.99 | 3.67 | 184.43 |
| CAT | Leaf | 2014 | 0.02 | 170.00 | 4.30 | 16.13 | 375.22 |
|  |  | 2015 | 0.01 | 512.81 | 10.36 | 53.06 | 512.30 |
|  | Root | 2014 | 0.00 | 95.00 | 6.69 | 15.80 | 236.08 |
|  |  | 2015 | 0.01 | 20.26 | 2.77 | 4.22 | 152.00 |
| proline | Leaf | 2014 | 0.27 | 18.75 | 2.91 | 2.89 | 99.24 |
|  |  | 2015 | 0.28 | 8.22 | 1.92 | 1.29 | 67.39 |
|  | Root | 2014 | 0.38 | 66.34 | 8.32 | 11.27 | 135.49 |
|  |  | 2015 | 0.26 | 26.66 | 4.58 | 4.83 | 105.35 |
