# Supplementary material for: Natural variation MeMYB108 associated with tolerance to stress-induced leaf abscission linked to enhanced protection against reactive oxygen species in cassava
Source: Plant Cell Rep. 2022 May 24;41(7):1573–87. doi: 10.1007/s00299-022-02879-6 (PMC9270272; doi:10.1007/s00299-022-02879-6)
Supplement: Supplementary file 4 — Supplementary file4 (DOCX 16 KB) [file 299_2022_2879_MOESM4_ESM.docx]

**Supplementary Table S4.** Distribution of nucleotide variants in *MeMYB108*

| Gene | Gene length | NO.of exons | Genomic region | | | Exon | | | Conserved Domain | |
| --- | --- | --- | --- | --- | --- | --- | --- | --- | --- | --- |
|  |  |  | SNP | Indel | Synonymous | Non-synonymous | Frame shift | Stop  gained | Synonymous | Non-synonymous |
| *MeMYB2* | 1027 | 3 | 87 | 2 | 17 | 42 |  |  | 2 | 12 |
