# Supplementary material for: Natural variation MeMYB108 associated with tolerance to stress-induced leaf abscission linked to enhanced protection against reactive oxygen species in cassava
Source: Plant Cell Rep. 2022 May 24;41(7):1573–87. doi: 10.1007/s00299-022-02879-6 (PMC9270272; doi:10.1007/s00299-022-02879-6)
Supplement: Supplementary file 5 — Supplementary file5 (DOCX 17 KB) [file 299_2022_2879_MOESM5_ESM.docx]

**Supplemental Table S5.** ANOVA analysis of leaf abscission induced by drought tolerance coefficient of single traits among haplotype groups

| **Traits** |  | **Sum of Square** | **F value** | **Significant Level** |
| --- | --- | --- | --- | --- |
| CAT-L-2014 | Among | 763.000 | 4.420 | .002 |
|  | Within | 5610.448 |  |  |
|  | Total | 6373.449 |  |  |
| Proline-L-2015 | Among | 20.613 | 2.643 | .038 |
|  | Within | 196.906 |  |  |
|  | Total | 217.520 |  |  |
| POD-R-2015 | Among | 162511.283 | 3.632 | .009 |
|  | Within | 1040164.070 |  |  |
|  | Total | 1202675.353 |  |  |
