# Supplementary material for: Natural variation MeMYB108 associated with tolerance to stress-induced leaf abscission linked to enhanced protection against reactive oxygen species in cassava
Source: Plant Cell Rep. 2022 May 24;41(7):1573–87. doi: 10.1007/s00299-022-02879-6 (PMC9270272; doi:10.1007/s00299-022-02879-6)
Supplement: Supplementary file 6 — Supplementary file6 (DOCX 22 KB) [file 299_2022_2879_MOESM6_ESM.docx]

**Supplemental Table S6.** The correlation analyses between *MeMYB108* and reactive oxygen scavengers under the leaf abscission exposure to drought in cassava

| **Traits** | **Locus** | **p_Marker** | **Rsq_marker** | **Annotation** |
| --- | --- | --- | --- | --- |
| CAT-L-2014 | SNP1_31465078 | 0.0192 | 0.0854 | Intron_Variant |
| CAT-L-2014 | SSR249 | 0.0192 | 0.0854 | Intron_Variant |
| CAT-L-2014 | SNP1_31465080 | 0.0167 | 0.0884 | Intron_Variant |
| CAT-L-2014 | SNP1_31465123 | 0.0192 | 0.0854 | Intron_Variant |
| CAT-L-2014 | SNP1_31465148 | 0.0205 | 0.084 | Intron_Variant |
| CAT-L-2014 | SNP1_31465183 | 0.0499 | 0.0657 | Synonmous_Variant |
| CAT-L-2014 | SNP1_31465184 | 0.0205 | 0.084 | Synonmous_Variant |
| CAT-L-2014 | SNP1_31465789 | 2.26E-05 | 0.232 | Misense_Variant |
| CAT-R-2014 | SNP1_31465813 | 0.0331 | 0.0672 | Misense_Variant |
| SOD-L-2014 | SNP1_31465012 | 0.0267 | 0.049 | Synonmous_Variant |
| SOD-L-2014 | SNP1_31465207 | 0.0468 | 0.0423 | Synonmous_Variant |
| SOD-L-2014 | SNP1_31465222 | 0.0468 | 0.0423 | Synonmous_Variant |
| SOD-L-2014 | SNP1_31465325 | 0.0488 | 0.0399 | Intron_Variant |
| AGFW-2014 | SNP1_31465789 | 0.0339 | 0.0922 | Misense_Variant |
| SRDMP-2014 | SNP1_31465842 | 4.70E-20 | 0.3914 | Synonmous_Variant |
| SRDMP-2014 | SNP1_31465847 | 4.70E-20 | 0.3914 | Misense_Variant |
| SRDW-2014 | SNP1_31465012 | 0.0026 | 0.1467 | Synonmous_Variant |
| SRDW-2014 | SNP1_31465078 | 0.037 | 0.0841 | Intron_Variant |
| SRDW-2014 | SSR249 | 0.037 | 0.0841 | Intron_Variant |
| SRDW-2014 | SNP1_31465080 | 0.037 | 0.0842 | Intron_Variant |
| SRDW-2014 | SNP1_31465123 | 0.037 | 0.0841 | Intron_Variant |
| SRDW-2014 | SNP1_31465148 | 0.037 | 0.0841 | Intron_Variant |
| SRDW-2014 | SNP1_31465184 | 0.037 | 0.0841 | Synonmous_Variant |
| SRN-2014 | SNP1_31465018 | 0.0145 | 0.0707 | Synonmous_Variant |
| SRN-2014 | SNP1_31465046 | 0.0145 | 0.0707 | Intron_Variant |
| SRN-2014 | SNP1_31465789 | 2.87E-04 | 0.2077 | Misense_Variant |
| SRFW-2014 | SNP1_31465012 | 0.0058 | 0.1286 | Synonmous_Variant |
| SRFW-2014 | SNP1_31465078 | 0.0411 | 0.0818 | Intron_Variant |
| SRFW-2014 | SSR249 | 0.0411 | 0.0818 | Intron_Variant |
| SRFW-2014 | SNP1_31465080 | 0.041 | 0.0819 | Intron_Variant |
| SRFW-2014 | SNP1_31465123 | 0.0411 | 0.0818 | Intron_Variant |
| SRFW-2014 | SNP1_31465148 | 0.0411 | 0.0818 | Intron_Variant |
| SRFW-2014 | SNP1_31465184 | 0.0411 | 0.0818 | Synonmous_Variant |
| SRS-R-2014 | SNP1_31465762 | 0.0159 | 0.0899 | Misense_Variant |
| SRS-R-2014 | SNP1_31465765 | 0.0159 | 0.0899 | Misense_Variant |
| SRS-R-2014 | SNP1_31465804 | 0.0159 | 0.0899 | Misense_Variant |
| SRS-R-2014 | SNP1_31465842 | 0.0145 | 0.0919 | Synonmous_Variant |
| SRS-R-2014 | SNP1_31465847 | 0.0145 | 0.0919 | Misense_Variant |
| Pro-L-2014 | SNP1_31465447 | 8.96E-04 | 0.0706 | Misense_Variant |
| CAT-L-2015 | SNP1_31465012 | 0.0468 | 0.0676 | Synonmous_Variant |
| CAT-L-2015 | SNP1_31465207 | 0.0468 | 0.0676 | Synonmous_Variant |
| CAT-L-2015 | SNP1_31465222 | 0.0468 | 0.0676 | Synonmous_Variant |
| CAT-R-2015 | SNP1_31465018 | 0.0229 | 0.0717 | Synonmous_Variant |
| CAT-R-2015 | SNP1_31465028 | 0.0303 | 0.0667 | Misense_Variant |
| CAT-R-2015 | SNP1_31465044 | 0.0303 | 0.0667 | Intron_Variant |
| CAT-R-2015 | SNP1_31465046 | 0.0229 | 0.0717 | Intron_Variant |
| CAT-R-2015 | SNP1_31465124 | 0.0303 | 0.0667 | Intron_Variant |
| CAT-R-2015 | SNP1_31465279 | 0.0303 | 0.0667 | Synonmous_Variant |
| CAT-R-2015 | SNP1_31465607 | 1.96E-04 | 0.2005 | Synonmous_Variant |
| CAT-R-2015 | SNP1_31465734 | 1.96E-04 | 0.2005 | Misense_Variant |
| CAT-R-2015 | SNP1_31465762 | 0.0024 | 0.1373 | Misense_Variant |
| CAT-R-2015 | SNP1_31465765 | 0.0024 | 0.1373 | Misense_Variant |
| CAT-R-2015 | SNP1_31465773 | 1.96E-04 | 0.2005 | Misense_Variant |
| CAT-R-2015 | SNP1_31465774 | 1.96E-04 | 0.2005 | Misense_Variant |
| CAT-R-2015 | SNP1_31465804 | 0.0024 | 0.1373 | Misense_Variant |
| CAT-R-2015 | SNP1_31465842 | 0.0067 | 0.1503 | Synonmous_Variant |
| CAT-R-2015 | SNP1_31465847 | 0.0067 | 0.1503 | Misense_Variant |
| MDA-R-2015 | SNP1_31465048 | 0.0355 | 0.0811 | Intron_Variant |
| POD-R-2015 | SNP1_31465012 | 0.042 | 0.0663 | Synonmous_Variant |
| POD-R-2015 | SNP1_31465207 | 0.042 | 0.0663 | Synonmous_Variant |
| POD-R-2015 | SNP1_31465222 | 0.042 | 0.0663 | Synonmous_Variant |
| Pro-L-2015 | SNP1_31465078 | 0.0341 | 0.0954 | Intron_Variant |
| Pro-L-2015 | SSR249 | 0.0341 | 0.0954 | Intron_Variant |
| Pro-L-2015 | SNP1_31465123 | 0.0341 | 0.0954 | Intron_Variant |
| Pro-L-2015 | SNP1_31465509 | 0.0424 | 0.0899 | Misense_Variant |
| SOD-L-2015 | SNP1_31465607 | 0.0446 | 0.0523 | Synonmous_Variant |
| SOD-L-2015 | SNP1_31465734 | 0.0446 | 0.0523 | Misense_Variant |
| SOD-L-2015 | SNP1_31465762 | 0.0394 | 0.0547 | Misense_Variant |
| SOD-L-2015 | SNP1_31465765 | 0.0394 | 0.0547 | Misense_Variant |
| SOD-L-2015 | SNP1_31465773 | 0.0446 | 0.0523 | Misense_Variant |
| SOD-L-2015 | SNP1_31465774 | 0.0446 | 0.0523 | Misense_Variant |
| SOD-L-2015 | SNP1_31465804 | 0.0394 | 0.0547 | Misense_Variant |
| SOD-R-2015 | SNP1_31465447 | 0.0227 | 0.1 | Misense_Variant |
| SRS-R-2015 | SNP1_31465789 | 1.50E-04 | 0.17 | Misense_Variant |
