# Supplementary material for: Natural variation MeMYB108 associated with tolerance to stress-induced leaf abscission linked to enhanced protection against reactive oxygen species in cassava
Source: Plant Cell Rep. 2022 May 24;41(7):1573–87. doi: 10.1007/s00299-022-02879-6 (PMC9270272; doi:10.1007/s00299-022-02879-6)
Supplement: Supplementary file 7 — Supplementary file7 (DOCX 17 KB) [file 299_2022_2879_MOESM7_ESM.docx]

**Supplemental Table S7.** Network regulation of *MeMYB108* and reactive oxygen scavengers encoding genes.

| New candidate pathway gene | | | Score | Evidence provided  by AraNet | Genes involved in functional network | |
| --- | --- | --- | --- | --- | --- | --- |
| Arabidopsis  ID | Gene  name | Cassava  orthology |  |  | Arabidopsis  orthology | Cassava candidate gene |
| AT4G12400 | Hop3 | 01G075100 | 13.01 | AT-CC:0.80  SC-CC:0.20 | AT1G07400;  MYB2; MYB15 | 02G124800; *MeMYB2*,  15G040700 |
| AT3G57530 | CPK32 | 09G024500 | 12.51 | AT-CC:0.51  AT-PG:0.49 | AT1G28390;  MYB2; MYB15;  AT4G2729 | 18G061500; *MeMYB2*;  15G040700; 03G119400 |
| AT4G21440 | MYB102 | 16G019700 | 11.74 | AT-CC:1.00 | AT1G07400;  MYB2; MYB15 | 02G124800; *MeMYB2*;  15G040700 |
| AT1G07400 |  | 02G124800 | 14.01 | SC-CC:0.69  AT-CC:0.20  SC-CX:0.11 | CZSOD2; MYB2;  MSD1;  AT3G56350 | 08G125400; *MeMYB2*;  07G140500 |
| AT4G21440 | MYB102 | 16G019700 | 8.77 | AT-CC:1.00 | MYB2; DHS1 | *MeMYB2*; 13G095700 |
| AT2G46270 | GBF3 | 01G249900 | 8.48 | AT-CC:1.00 | MYB2; DHS1 | *MeMYB2*; 13G095700 |
