# Supplementary material for: Natural variation MeMYB108 associated with tolerance to stress-induced leaf abscission linked to enhanced protection against reactive oxygen species in cassava
Source: Plant Cell Rep. 2022 May 24;41(7):1573–87. doi: 10.1007/s00299-022-02879-6 (PMC9270272; doi:10.1007/s00299-022-02879-6)
Supplement: Supplementary file 8 — Supplementary file8 (DOCX 4029 KB) [file 299_2022_2879_MOESM8_ESM.docx]

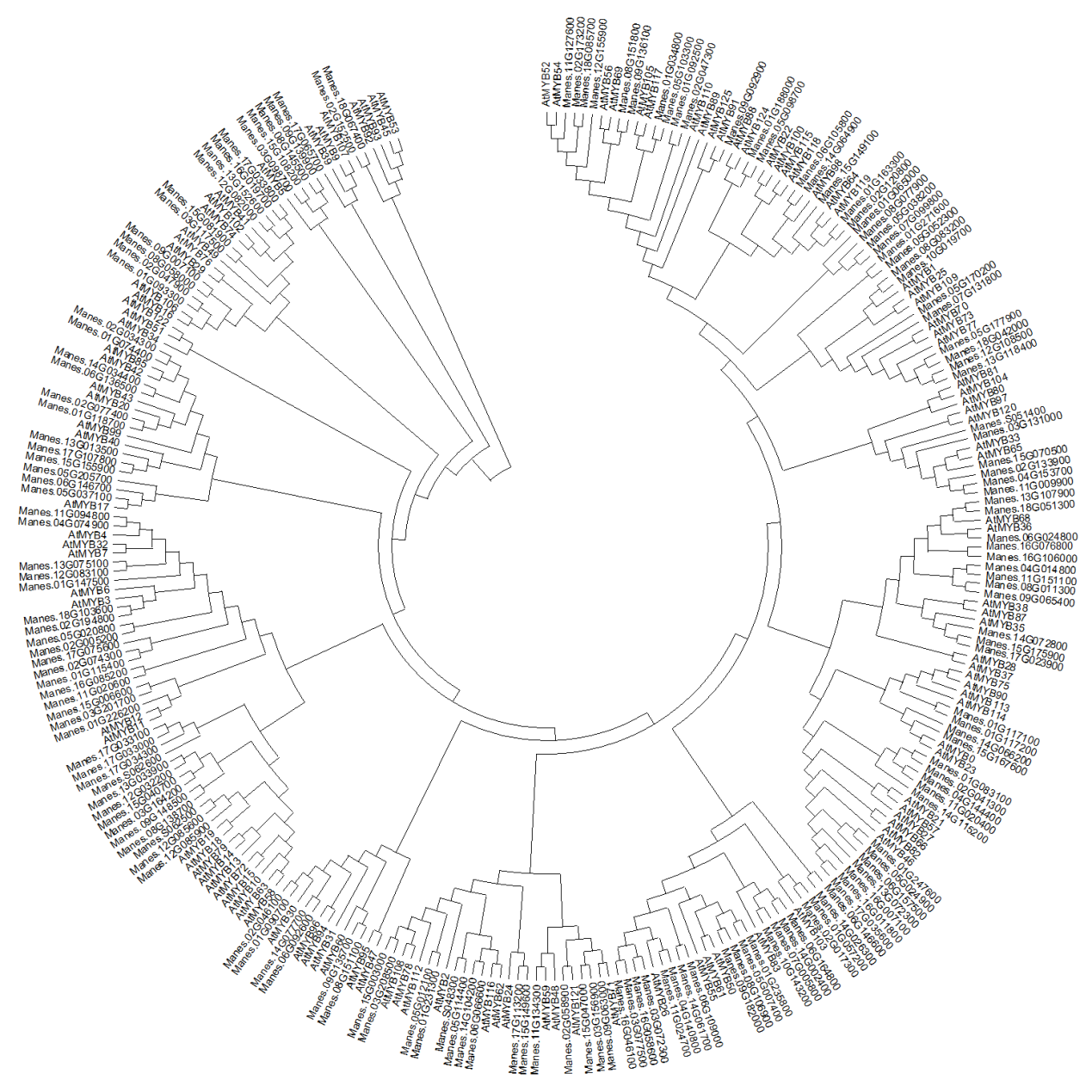


a

b


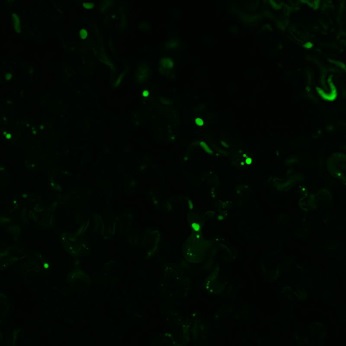

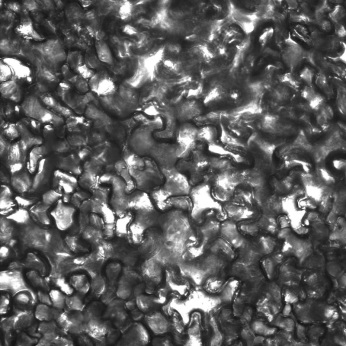

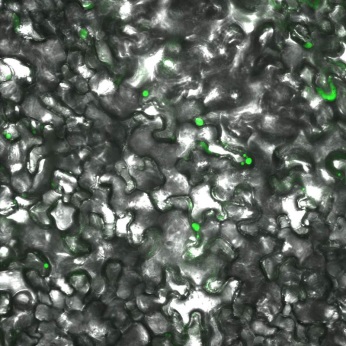


GFP Bright field Merged

**Supplementary Fig. 1** **a** *MeMYB108* encodes a *MYB* transcription factor. Phylogenetic analysis of the *MYB* transcription factors of Arabidopsis and cassava. Eighbor-joining phylogeny of *MYB* genes of 2 species, as determined by MEGA5.0. Numbers on branches are bootstrap proportions from 1000 replicates. The Red box shows *MeMYB108*. **b** Subcellular localization of *MeMYB108* in tobacco epidermal cells.

a


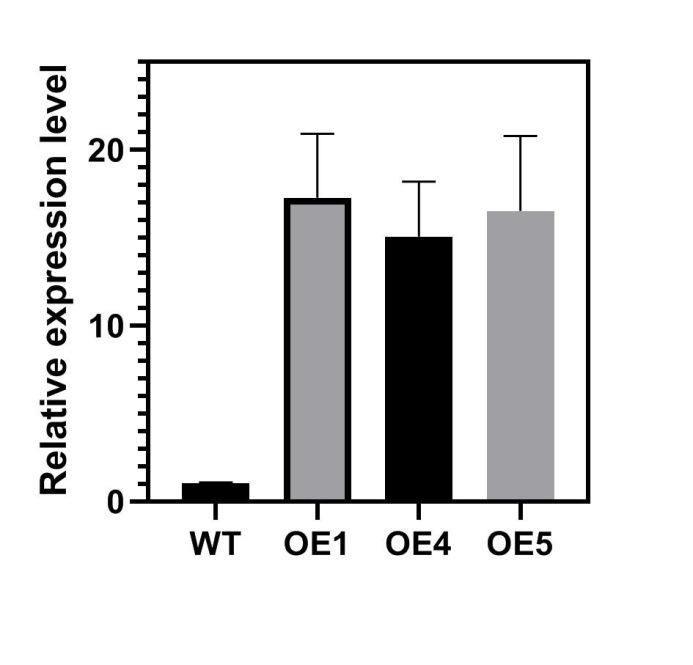


b


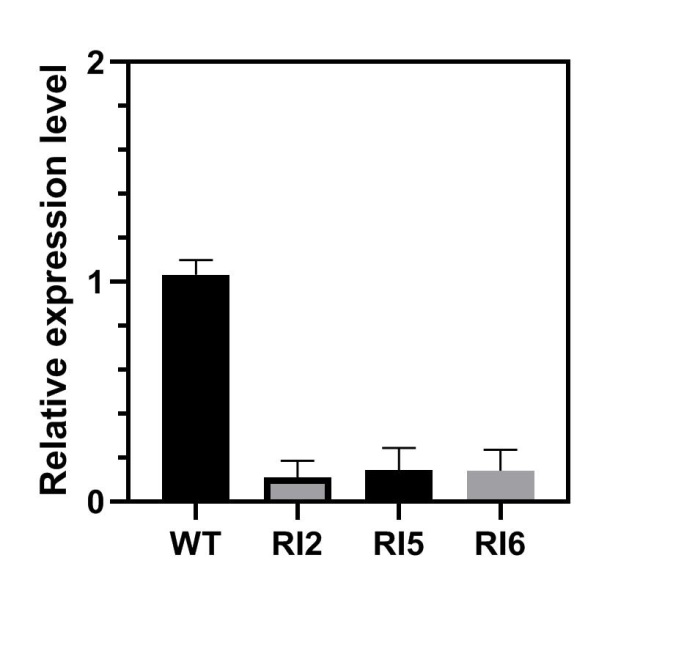


d

c


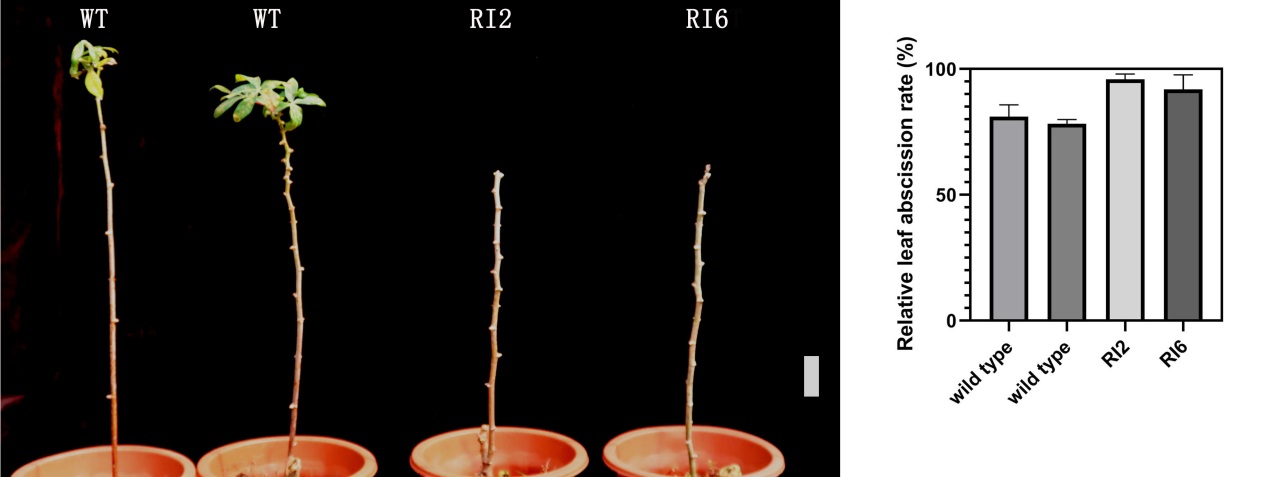


**Supplementary Fig. 2** The expression levels of *MeMYB108* in wildtype, overexpression lines (OE1, OE4, OE5) (a) and *MeMYB108*-RNA interference lines (RI2, RI5, RI6) (b) were analyzed by RT-qPCR. Error bars indicate the SE based on three technical replicates. Two transgenic lines OE1 and OE5, and two RNA interference transgenic lines RI2 and RI6 were selected for futher analysis. **c** Comparsion of *MeMYB108*-RNA interference lines and wild type plants were subjected to MLA conditions without being watered for 14 days and then recovered for 7 days. Bars=50 mm. **d** Leaf abscission rates of transgenic and wild-type plants tested in c. Values are means±SE (n=4). Statistical significance was determined by Student’s t test.


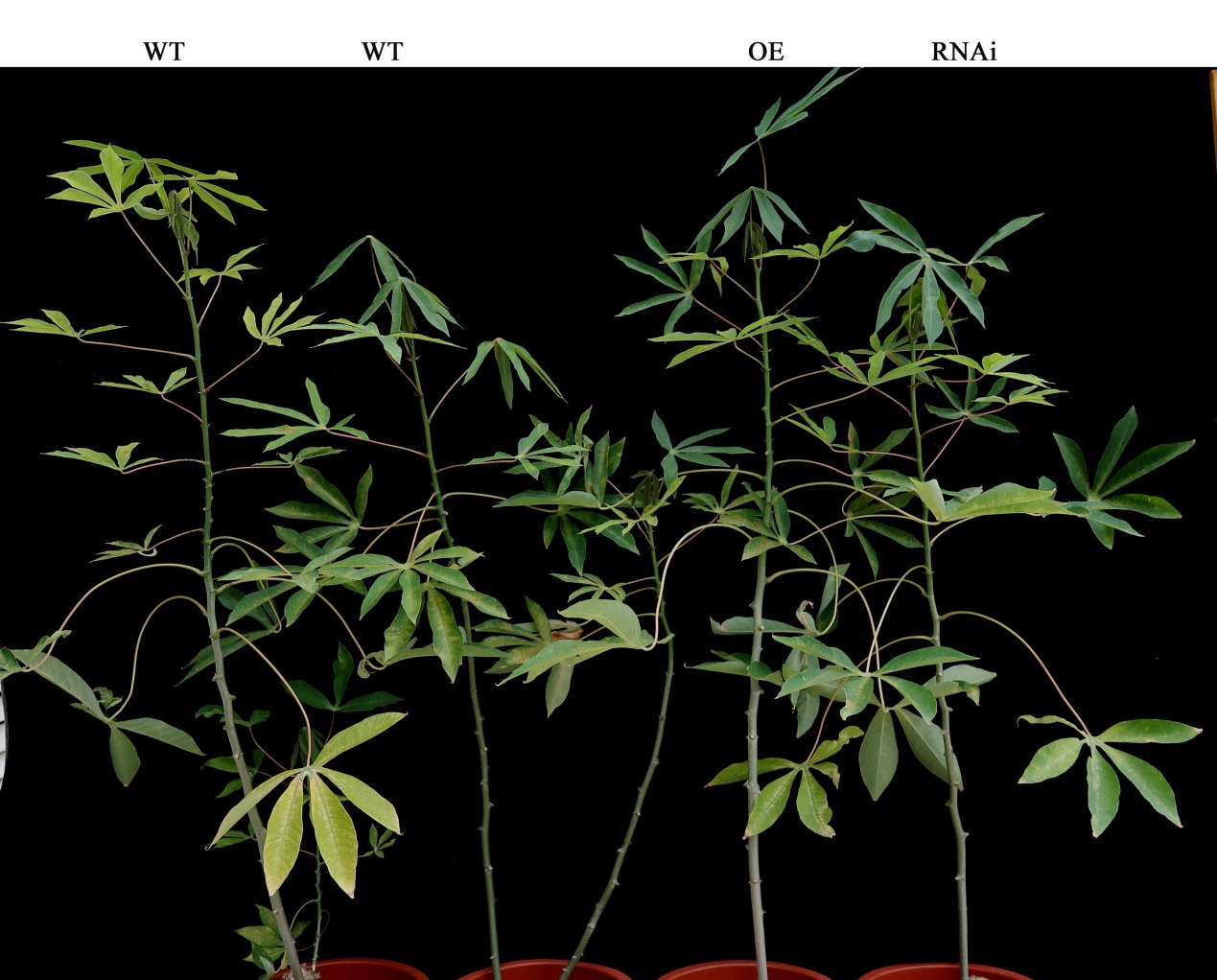


**Supplementary Fig. 3** Phenotype of WT, *MeMYB108-*OE and *MeMYB108*-RNAi plants under the same normal growth conditions


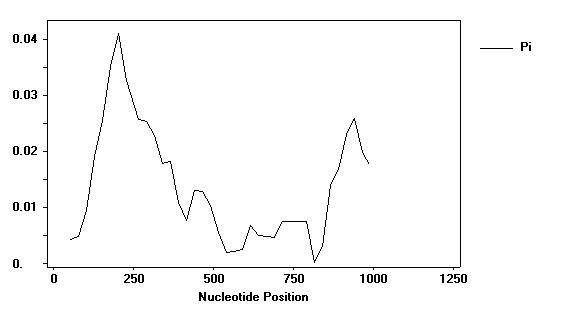


**Supplementary Fig. 4** Nucleotide diversity of *MeMYB108* by sliding windows analysis.

The DNA nucleotide diversity was analyzed by sliding window with a window size of 100bp and a step size of 25bp using DnaSP5 software


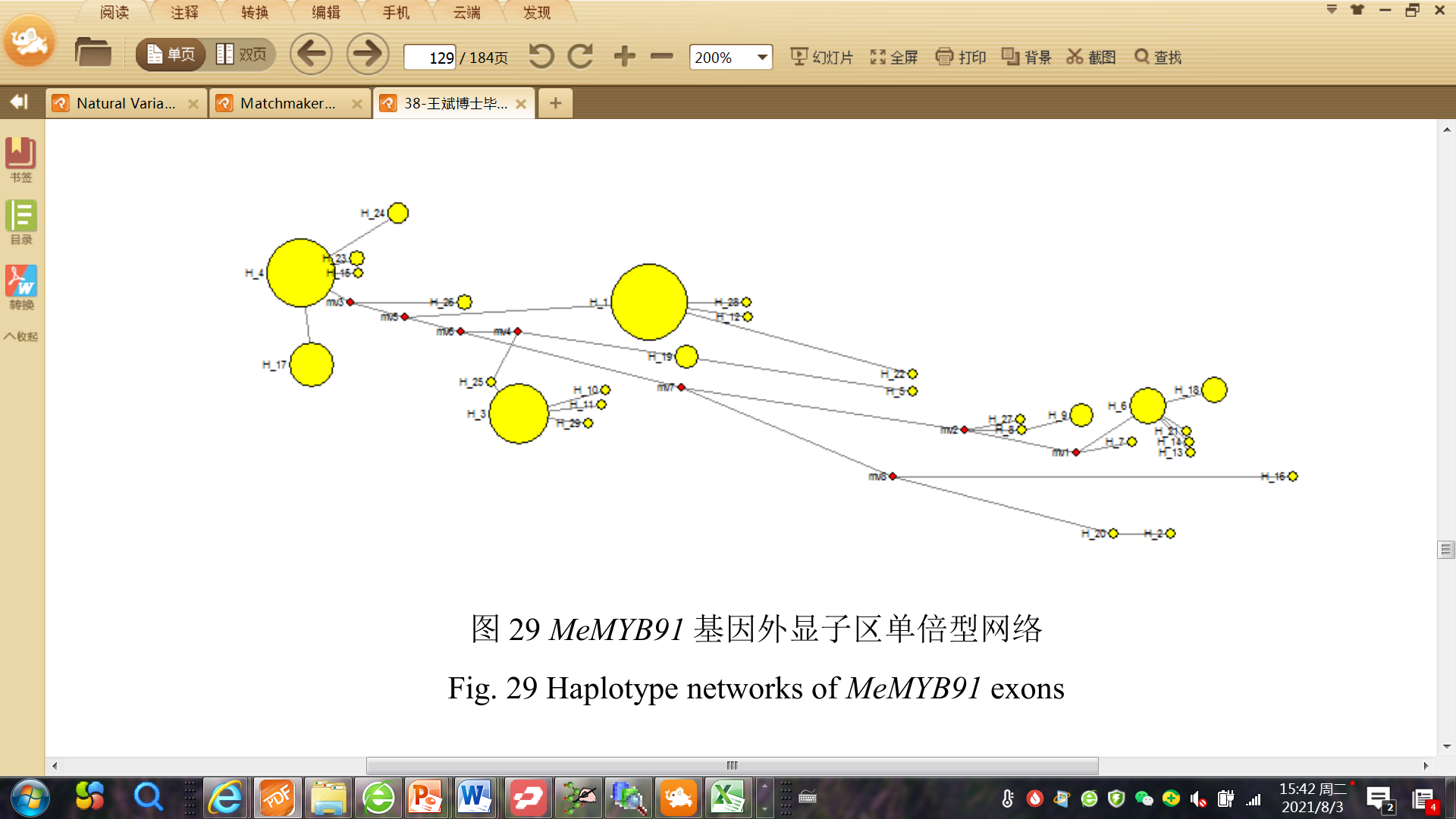


**Supplementary Fig. 5** Haplotype networks of *MeMYB91* exons.

The exon region of the *MeMYB108* gene includes 29 haplotypes and 7 major haplotypes.


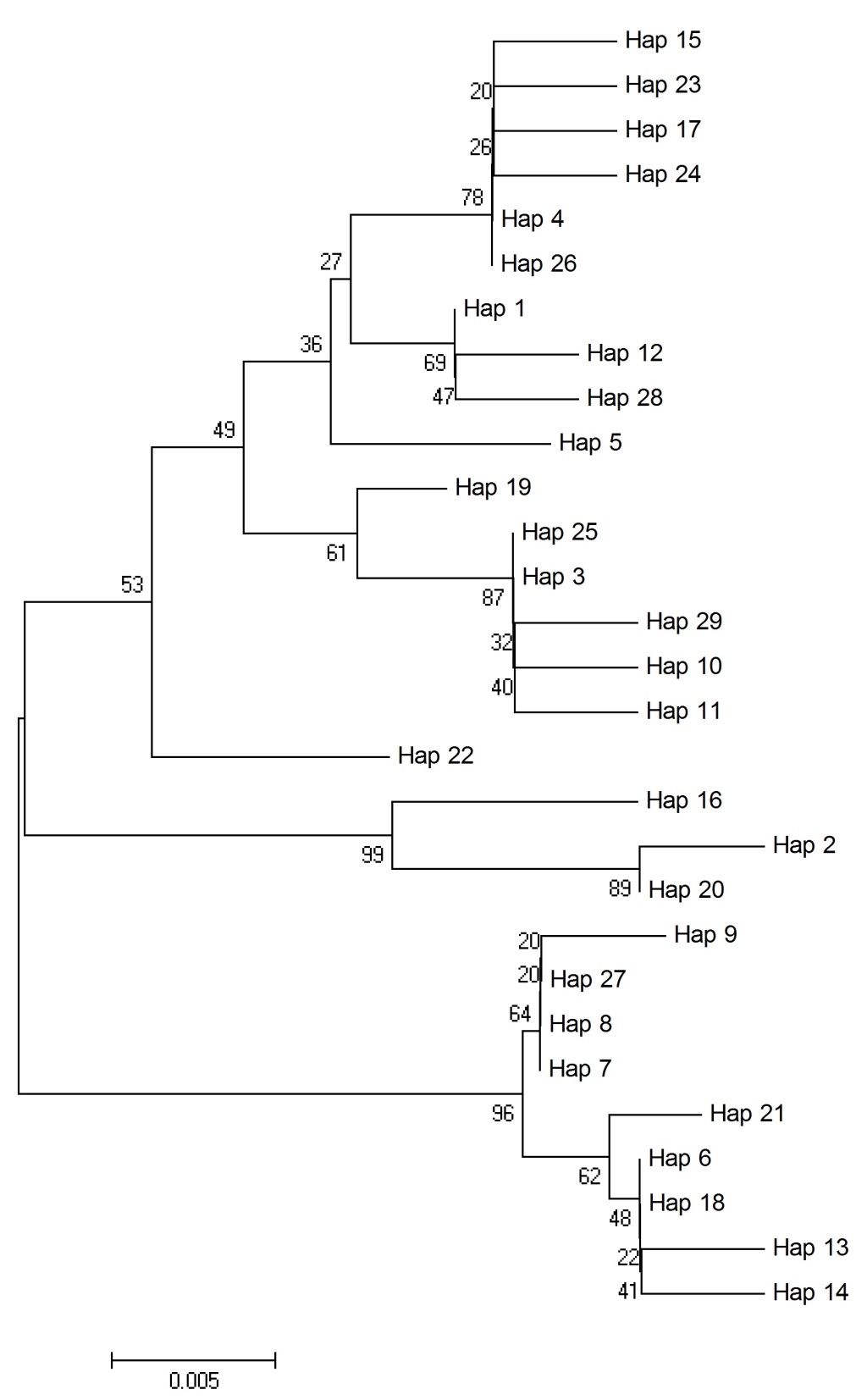


**Supplementary Fig.6** Haplotypes phylogenetic tree of *MeMYB108* exons. Neighbor-joining phylogenetic analysis is shown for the *MeMYB108* exons. The phylogenetic tree was constructed using the ClustalW and MEGA programs. Tree topology with bootstrap support is based on a percentage of 1,000 replicates. Those numbers on the nodes are bootstrap percentages, indicating the reliability of the cluster descending from that node. All of these haplotypes can be divided into two categories.


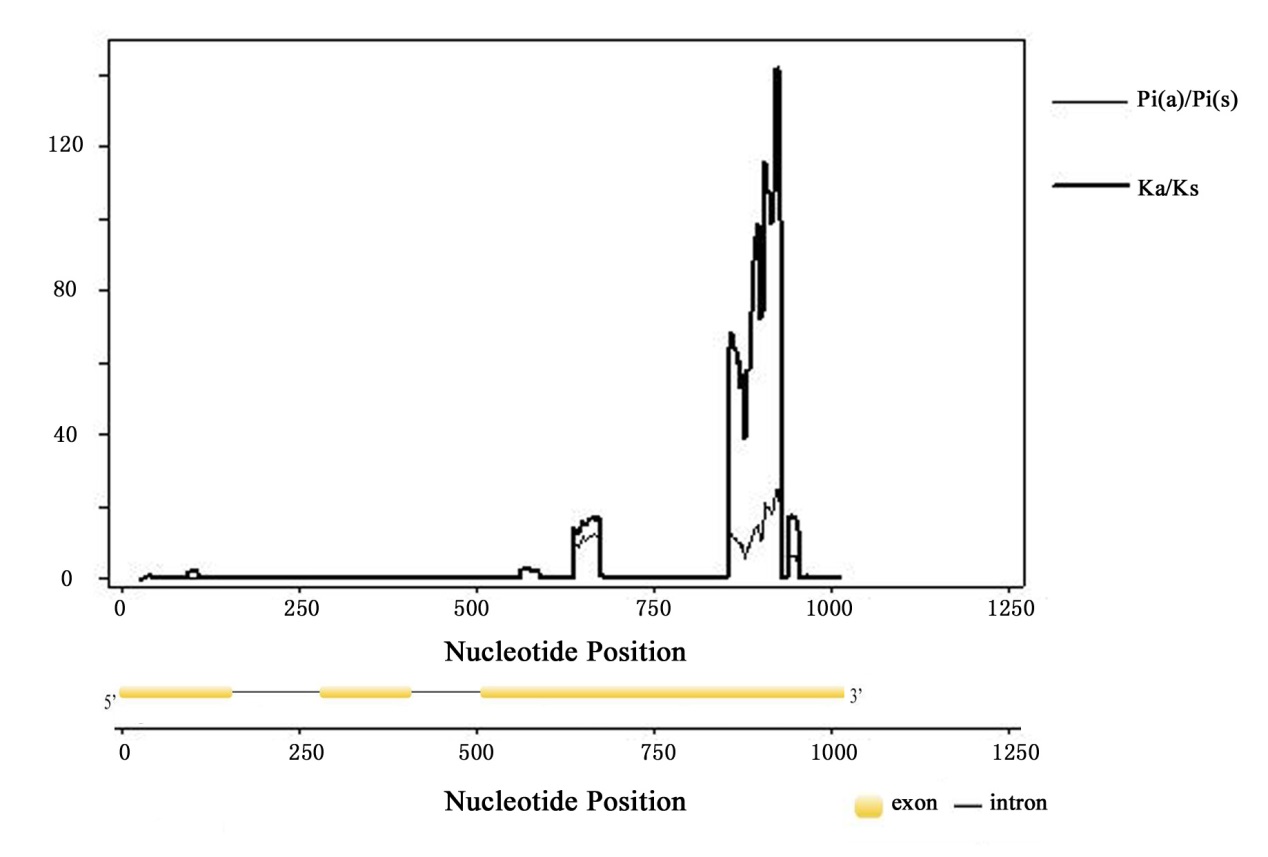


Exon 3

Exon 2

Exon 1

**Supplementary Fig.7** Ka/Ks analysis of two cluster exon haplotypes of *MeMYB108* by sliding windows method. Positive selection was indicated in the third exon region.

Note：Nucleotide position means the location which was relative to the first nucleotide A of the start codon.


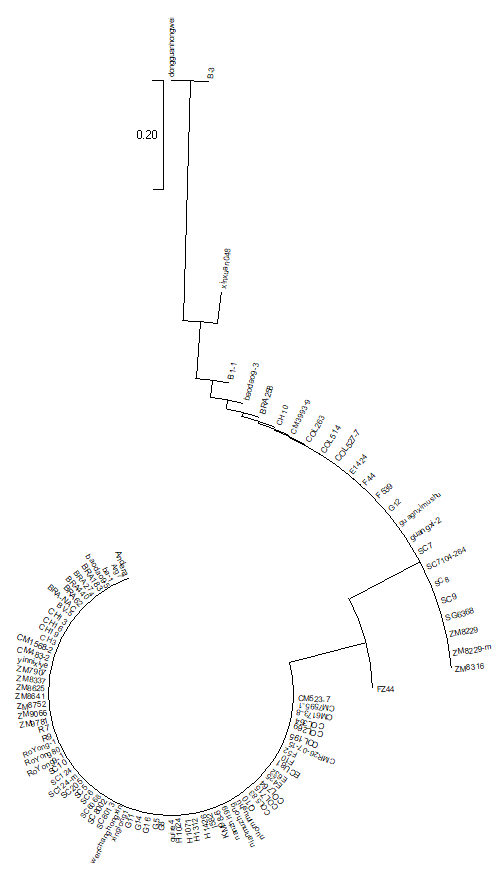


| 31465734 | 31465762 | 31465765 | 31465773 | 31465774 | 31465804 |  |
| --- | --- | --- | --- | --- | --- | --- |
| S1 | S2 | S3 | S4 | S5 | S6 | Frequency |
| G | G | C | G | G | G | 0.853 |
| A | A | A | T | T | A | 0.134 |
| A | G | C | T | T | G | 0.013 |

**Supplementary Fig. 8** Phylogenetic tree of *MeMYB108* in 97 varieties constructed based on seven SNPs (S1–S6).

Six SNPs, SNP1_31465734, SNP1_31465773, SNP1_31465774, SNP1_31465762, SNP1_31465765 and SNP1_31465804, were significantly associated with CAT under leaf abscission induced by drought. The NJ cluster tree of 97 cassava varieties/lines was constructed based on the six SNPs, and 97 cassava accessions were divided into two groups. There was significant linkage disequilibrium among the six SNPs.
